# COX2 ^+^/PDGFRα ^+^ fibroblasts selectively localize near bile ducts and interact with immune cells in early liver fibrosis in mice

**DOI:** 10.14814/phy2.70938

**Published:** 2026-05-24

**Authors:** Momo Goto, Tamaki Kurosawa, Hiroyuki Koike, Noriyuki Kaji, Ichiro Manabe, Taiki Mihara, Takashi Chaen, Yumiko Oishi, Masatoshi Hori

**Affiliations:** ^1^ Laboratory of Veterinary Pharmacology, Department of Veterinary Medical Sciences, Graduate School of Agriculture and Life Sciences The University of Tokyo Bunkyo‐ku Tokyo Japan; ^2^ Department of Medical Biochemistry, Graduate School of Medical and Dental Sciences Institute of Science Tokyo Bunkyo‐ku Tokyo Japan; ^3^ Laboratory of Veterinary Pharmacology, School of Veterinary Medicine Azabu University Sagamihara Kanagawa Japan; ^4^ Department of Systems Medicine, Graduate School of Medicine Chiba University Chiba Chiba Japan

**Keywords:** COX2, fibroblast, liver fibrosis, PDGFRα, single cell RNA sequencing

## Abstract

Liver fibrosis is a growing global health problem with no effective treatments. PDGFRα‐positive fibroblasts are the main drivers of liver fibrosis, but their cellular heterogeneity is not fully understood. In this study, we performed single‐cell RNA sequencing (scRNA‐seq) on PDGFRα‐positive fibroblasts isolated from healthy mouse livers. We also induced liver fibrosis via bile duct ligation (BDL) and examined their interactions with immune cells. scRNA‐seq revealed that PDGFRα‐positive fibroblasts mainly consist of hepatic stellate cells and portal fibroblasts. CellChat analysis indicated that a COX2‐high (*Ptgs2*‐high) subpopulation may preferentially interact with immune cells. This COX2‐positive PDGFRα‐positive fibroblast subset, also reported in other organs, localizes around bile ducts in the liver. Furthermore, when FACS‐sorted PDGFRα‐positive fibroblasts were treated with IL‐1β in vitro, the proportion of COX2‐positive cells increased. These findings demonstrate that PDGFRα‐positive fibroblasts exhibit diversity in their responses to immune signals, with the COX2‐positive subset potentially playing a key role in early fibrotic responses. Clarifying this heterogeneity may improve understanding of early liver fibrosis and support development of new therapeutic strategies.

## Graphical abstract

COX2^+^PDGFRα^+^ fibroblasts show selective localization and immune crosstalk in bile duct ligation‐induced liver fibrosis.

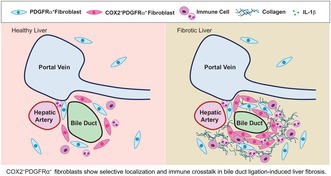

## 1 INTRODUCTION

Liver fibrosis is a progressive condition characterized by the activation of fibroblasts in the liver interstitium in response to tissue injury from infections or worsening metabolic conditions. These activated fibroblasts produce excessive extracellular matrix (ECM), which, when excessively deposited, compresses the liver parenchyma and impairs its function (Bataller & Brenner, 2005; Rockey et al., 2015). Liver fibrosis remains reversible up to a critical threshold. If ECM accumulation is mild, addressing the underlying cause of tissue injury can promote the breakdown of excess ECM and induce apoptosis of activated fibroblasts, facilitating healthy tissue regeneration. However, excessive ECM accumulation outpaces its degradation, leading to progression to cirrhosis (Iredale et al., 1998; Pellicoro et al., 2014). A 2024 meta‐analysis estimated the global prevalence of cirrhosis at 1.3%, with its precursor, liver fibrosis, reaching a prevalence of up to 3.3% (Zamani et al., 2024). Despite its severity, causing numerous complications and potential progression to liver cancer, no effective treatment for cirrhosis currently exists (Bataller & Brenner, 2005).

Developing effective treatments for liver fibrosis necessitates a thorough understanding of the cells driving fibrogenesis. Until around 2014, hepatic stellate cells (HSCs), the primary resident fibroblasts in the liver, were a major focus of liver fibrosis research, with PDGFRβ, an isoform of the platelet‐derived growth factor receptor (PDGFR) tyrosine kinase receptor family, used as a marker in numerous studies (Henderson et al., 2013; Kocabayoglu et al., 2015; Tsuchida & Friedman, 2017). Since 2019, PDGFRα, another PDGFR isoform, has been identified as a marker for cells contributing to fibrosis in the human liver (Ramachandran, Dobie, Wilson‐Kanamori, et al., 2019). In 2020, research using *Pdgfra* floxed and *Lrat*‐Cre mice demonstrated that hepatic stellate cell‐specific PDGFRα deficiency suppressed liver fibrosis (Kikuchi et al., 2020). In 2022, cells expressing PDGFRα, CD34, and CD9, with low CD200 expression, were identified as precursors of portal myofibroblasts (Lei et al., 2022). These findings highlight the critical role of PDGFRα‐positive cells in the pathogenesis of liver fibrosis. However, while PDGFRα‐positive cells include hepatic stellate cells and portal myofibroblasts (Kikuchi et al., 2020; Lei et al., 2022), their population composition, proportional distribution, and potential inclusion of other cell types remain poorly understood.

Immune cells also play a significant role in the progression and persistence of liver fibrosis. Chemokines, which regulate leukocyte migration, are known to exacerbate fibrotic pathology. Deficiency in chemokines such as *Ccl3* or *Ccl5*, or their receptor *Ccr2*, reduces inflammatory cell infiltration in liver fibrosis model mice, thereby mitigating fibrosis (Berres et al., 2010; Heinrichs et al., 2013; Mitchell et al., 2009). Furthermore, liver macrophages inhibit apoptosis of activated HSCs via the NF‐κB pathway, worsening fibrosis (Pradere et al., 2013). Notably, the liver exhibits sinusoidal flow from the portal vein to the central vein, forming distinct zones of immune response, and in injured livers, the accumulation of chemokines and inflammatory cells in specific tissue regions has been reported (Konishi et al., 2019; Miyamoto et al., 2024). As noted earlier, PDGFRα‐positive fibroblasts, which are implicated in fibrosis initiation, comprise multiple cell types. Some of these cells may interact with immune cells, but studies exploring such interactions are currently limited.

Single‐cell RNA sequencing (scRNA‐seq) of human livers conducted in 2019 identified PDGFRα‐positive cells as the primary drivers of fibrosis, revealing that this cell population consists of multiple subclusters. The study also suggested that cytokines, such as TNF and IL‐1β, secreted by macrophages may activate these cells, while PDGFRα‐positive cells may secrete chemokines to recruit immune cells (Ramachandran, Dobie, Wilson‐Kanamori, Dora, et al., 2019). Several scRNA‐seq studies on the liver have been reported, including those using liver fibrosis model mice and analyses that enriched fibroblasts through genetically modified mice designed to label key fibroblast marker genes, such as *Acta2*, *Col1a1*, or *Pdgfrb* (Su et al., 2021) (Dobie et al., 2019). However, as hepatocytes constitute 70%–80% of liver cells, characterizing fibroblast populations using scRNA‐seq data from whole liver tissue has significant limitations. Notably, PDGFRα‐positive cells account for approximately 1% of the total liver cell population (Kurosawa et al., 2024), and no studies have yet reported the selective isolation of these cells for scRNA‐seq analysis.

In this study, we analyzed PDGFRα‐positive cell populations using a mouse model of liver fibrosis induced by bile duct ligation. We focused on the relationship between PDGFRα‐positive cells and immune cells. scRNA‐seq of PDGFRα‐positive fibroblasts in healthy livers confirmed that this cell population primarily consists of portal myofibroblasts and hepatic stellate cells, as defined by established marker genes. These cell populations may be further classified based on their differential reactivity with immune cells. We identified COX2‐positive PDGFRα‐positive fibroblasts around bile ducts, which may selectively interact with immune cells, and demonstrated that these cells may increase in response to IL‐1β produced by immune cells. These findings suggest that PDGFRα‐positive fibroblasts within the liver niche function selectively during the progression of liver fibrosis, highlighting the importance of elucidating their cellular characteristics to understand the mechanisms underlying liver fibrosis.

## 2 RESULTS

### 2.1 Distribution changes of PDGFRα‐positive and CD45‐positive cells during bile duct ligation‐induced fibrosis

**FIGURE 1:**
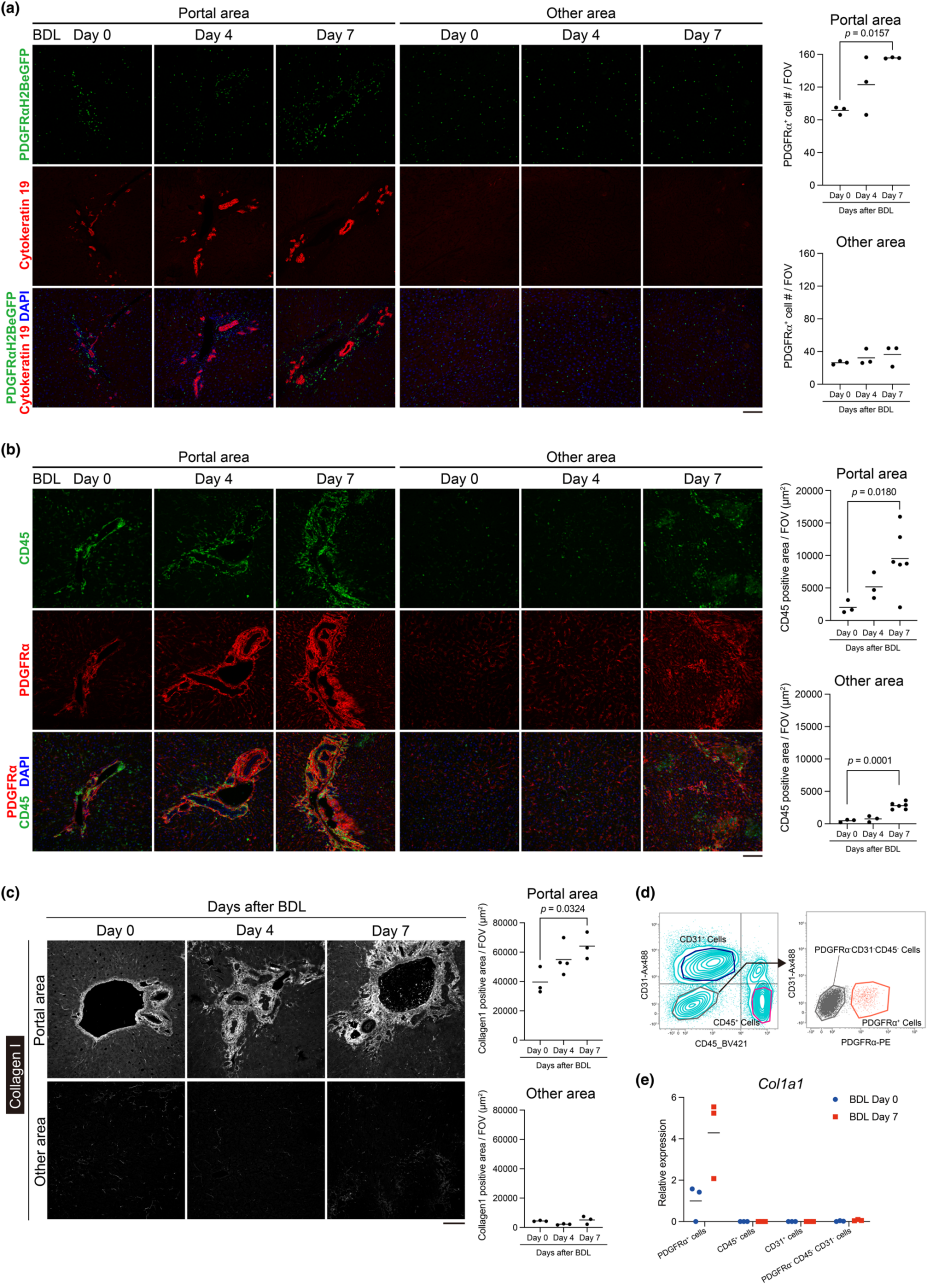
The BDL operation increases PDGFRα‐positive cells and CD45‐positive cells and causes the accumulation of collagen around the bile ducts. (a) Images of frozen sections from PDGFRα^EGFP^ mouse livers stained for cytokeratin 19 (red) and DAPI (blue) after the BDL operation. Cytokeratin 19 is used as a marker for bile ducts. The graphs show the number of eGFP‐positive cells in the field of view (FOV). (b) Images of frozen sections from WT mouse livers stained for CD45 (green), PDGFRα (red), and DAPI (blue). CD45 is used as a marker for pan‐hematopoietic cells. The graphs show the quantification of the CD45‐positive area in the field of view (day 0 and day 4, *n* = 3; day 7, *n* = 6). (c) Images of frozen sections from WT mouse livers stained for collagen type 1 (white) after the BDL operation. The graphs show the quantification of collagen type 1‐positive area in the field of view. Collagen type 1 is used as a marker for fibrotic areas. (d) The FACS plot shows the gating strategy for sorting CD31‐positive cells, CD45‐positive cells, PDGFRα‐positive cells, and CD31‐negative CD45‐negative PDGFRα‐negative cells from the livers of BDL Day 0 mice. (e) Real‐time PCR shows the relative expression levels of *Col1a1* in each cell. Each dot represents one mouse (a–c and e). For each mouse, two different fields from tissue sections were imaged and averaged to obtain a single value, which is shown as one dot (a–c). The bars in the graphs represent the mean value of the samples. Scale bar = 100 μm (a–c).

Previous studies in humans and mice have suggested that PDGFRα‐positive cells are a primary source of liver fibrosis (Kikuchi et al., 2020; Lei et al., 2022; Ramachandran, Dobie, Wilson‐Kanamori, et al., 2019). We investigated the dynamics and spatial distribution of these cells during the early stages of liver fibrosis. Using PDGFRα^EGFP^ mice, in which the nuclei of PDGFRα‐positive cells are labeled with GFP, we induced liver fibrosis via bile duct ligation (BDL) and analyzed the distribution of these cells at Days 0, 4, and 7 post‐surgery. Immunostaining revealed that PDGFRα‐positive cells were already abundant around bile ducts at day 0 before BDL (Figure 1a). By Day 7 post‐BDL, a significant increase in PDGFRα‐positive cells was observed predominantly around bile ducts (Figure 1a, left), whereas no significant increase was detected in other liver regions (Figure 1a, right). To assess the inflammatory response, we examined the distribution of CD45‐positive immune cells in wild‐type mice subjected to BDL. Immunostaining showed that these cells were already abundant around bile ducts at Day 0 before BDL (Figure 1b). By Day 7 post‐BDL, CD45‐positive cells significantly increased throughout the liver, with a particularly pronounced presence around bile ducts (Figure 1b). Notably, PDGFRα^EGFP^ mice exhibit reduced PDGFRα expression, and previous studies have shown that they develop attenuated fibrosis compared to wild‐type mice (Hayes et al., 2014). To verify that the restricted distribution observed in PDGFRα^EGFP^ mice was not an artifact of this reduced expression, we quantified the area of PDGFRα‐positive cells in wild‐type mice using immunostaining. Quantification of PDGFRα‐positive cell area in wild‐type mice yielded results generally consistent with those observed in PDGFR^αEGFP^ mice; however, in wild‐type mice, a significant increase in PDGFRα‐positive cells was also detected in non–peri‐biliary regions at Day 7 (Figure S1). To evaluate the regional progression of fibrosis, we assessed the distribution of type I collagen, a marker of fibrosis, in wild‐type mice subjected to BDL. Immunostaining revealed a significant increase in collagen accumulation predominantly around bile ducts at Day 7 post‐BDL, with no significant increase in other regions (Figure 1c). Using cell sorting to identify the source of collagen production, we found that cells highly expressing *Col1a1*, the gene encoding type I collagen, at day 7 post‐BDL were exclusively PDGFRα‐positive (Figure 1d,e). These data demonstrate that PDGFRα‐positive cells and CD45‐positive immune cells are predominantly localized in peri‐biliary regions in the liver following BDL. Consistent with previous reports, PDGFRα‐positive cells serve as the primary source of type I collagen production in liver fibrosis. Furthermore, during the early stages of BDL‐induced liver fibrosis, fibrogenesis is predominantly observed in peri‐biliary regions, consistent with the site of injury in this model.

### 2.2 Investigation of crosstalk between PDGFRα‐positive and CD45‐positive cells in fibrotic liver

**FIGURE 2:**
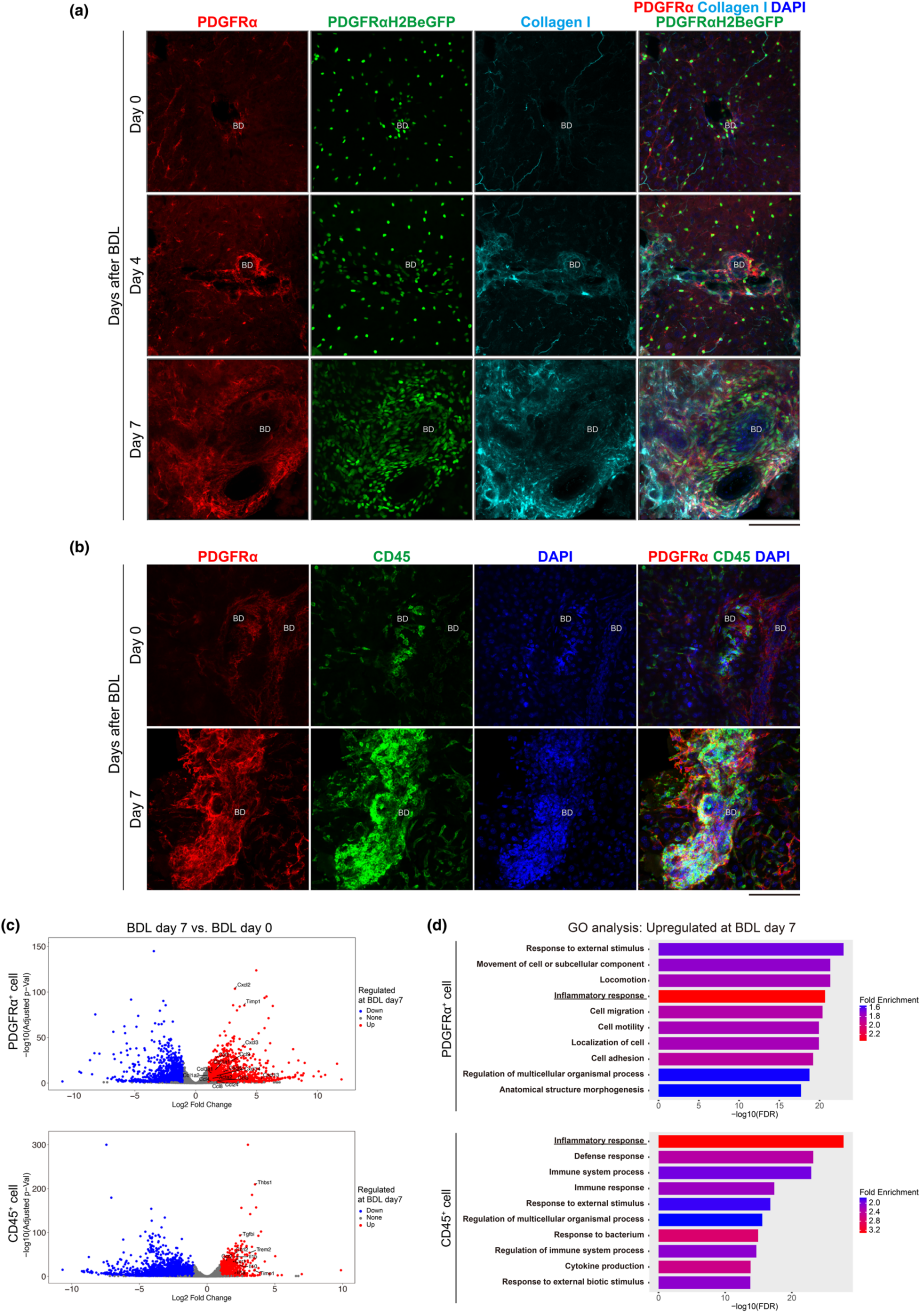
PDGFRα‐positive cells and CD45‐positive cells may interact with each other in fibrosis. (a) 3D images of whole‐mount PDGFRα^EGFP^ mouse livers stained for PDGFR (red), collagen type 1 (cyan), and DAPI (blue) after the BDL operation. BD in the figure indicates the bile duct. (b) 3D images of whole‐mount B6 mouse livers stained for PDGFR (red), CD45 (green), and DAPI (blue) after the BDL operation. (c) The results of bulk RNA‐seq DEG analysis between cells from mouse livers post BDL operation day 7 versus day 0. The volcano plot shows the DEGs in CD45‐positive cells and PDGFRα‐positive cells of mouse day 7 after BDL compared to that of day 0. DEGs were identified using DESeq2 with an FDR cutoff of 0.1 and a minimum fold‐change of 2, followed by the generation of volcano plots and GO analysis. (d) The bar plot shows the results of GO enrichment analysis. Scale bar = 100 μm (a, b).

The results shown in Figure 1 revealed that PDGFRα‐positive cells and CD45‐positive immune cells are closely co‐localized around bile ducts in the liver. To further examine their positional relationship, we performed whole‐mount staining of the liver and analyzed it using 3D imaging. The results showed that, during the early stages of fibrosis, the locations of PDGFRα‐positive cells around bile ducts overlapped with type I collagen‐positive regions (Figure 2a). At Day 7 post‐bile duct ligation (BDL), type I collagen was deposited in a manner that enveloped the regions containing PDGFRα‐positive cells (Figure 2a). In the liver at Day 7 post‐BDL, PDGFRα‐positive cells formed a dense, cage‐like structure, within which a large accumulation of CD45‐positive cells was observed (Figure 2b). These findings suggest that PDGFRα‐positive cells aggregate in specific areas, such as around bile ducts, during the early stages of liver fibrosis, producing abundant extracellular matrix (ECM) and potentially enhancing and stabilizing a niche for crosstalk with immune cells. To investigate the presence of crosstalk between PDGFRα‐positive and CD45‐positive cells, we performed bulk RNA sequencing on these cell populations at days 0 and 7 post‐BDL. DESeq2 analysis identified differentially expressed genes (DEGs), which are visualized in Figure 2c and listed in Tables S1 and S2. Notably, established markers of fibroblast activation and fibrogenesis, including *Acta2, Col1a1, Col1a2, Col3a1* and *Timp1*, were upregulated in PDGFRα‐positive cells at day 7 post‐BDL, supporting the validity of the BDL fibrosis model used in this study. In PDGFRα‐positive cells at day 7 post‐BDL, the expression of multiple genes encoding chemokines that regulate immune cell migration was upregulated, including *Cxcl1* (Figure S2), *Cxcl2*, *Cxcl3*, *Cxcl5*, *Cxcl13*, *Cxcl14*, *Ccl2*, *Ccl3*, *Ccl4*, *Ccl8*, *Ccl9*, and *Ccl24* (Figure 2c, upper). In CD45‐positive cells at day 7 post‐BDL, upregulated genes included those encoding chemokine receptors, such as *Ccr1* and *Ccr2* (Figure 2c, lower). The expression level of *Cxcr2*, which encodes a receptor for CXCL1 and CXCL2, was maintained in CD45‐positive cells at day 7 post‐BDL (Figure S2). Furthermore, CD45‐positive cells at Day 7 post‐BDL showed increased expression of genes encoding secreted proteins known to regulate fibrosis by acting on fibroblasts, including *Thbs1*, *Tgfbi*, *Trem2*, *Il1b*, *Il1a*, and *Il10* (Figure 2c, lower). Gene Ontology (GO) enrichment analysis of DEGs further indicated that the inflammatory response was the top enriched term for both cell types at day 7 post‐BDL (Figure 2d). These results demonstrate that PDGFRα‐positive and CD45‐positive cells are closely associated within collagen‐encased structures during the early stages of liver fibrosis. During disease progression, both cell types significantly upregulate genes encoding complementary ligands and receptors, suggesting enhanced crosstalk between them.

### 2.3 Exploration of PDGFRα‐positive cell subtypes in the liver using single‐cell RNA sequencing

**FIGURE 3:**
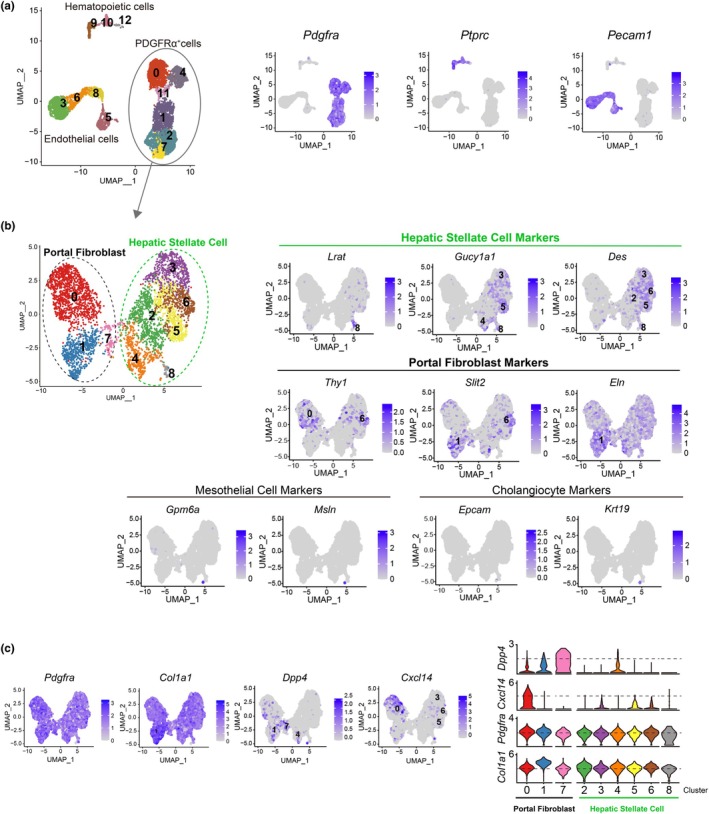
Single‐cell RNA sequencing reveals that PDGFRα‐positive cells include both hepatic stellate cells and portal fibroblasts. (a) UMAP plot of single‐cell RNA sequencing of sorted liver cells (left). Feature plots for *Pdgfra*, *Ptprc*, and *Pecam1* (right). (b) UMAP plot shows only PDGFRα‐positive cells extracted from (a) (left). Feature plots display the expression of marker genes for hepatic stellate cell, portal fibroblast, mesothelial cell, and cholangiocyte (right). (c) Feature plots (left) and violin plots (right) display the expression of *Pdgfra*, *Col1a1*, *Dpp4*, and *Cxcl14* in PDGFRα‐positive cells.

Hepatic stellate cells (HSCs) and portal fibroblasts (PFs) located near the portal vein are well‐known as the primary fibroblast populations in the liver (Iwaisako et al., 2014; Wells, 2014). Additionally, mesothelial cells, cholangiocytes, and lymphatic endothelial cells have been suggested as potential progenitor cells for myofibroblasts in the liver (Huang et al., 2023; Li et al., 2013; Yu et al., 2018). To investigate the composition of PDGFRα‐positive cell populations, we performed scRNA‐seq on PDGFRα‐positive cells, endothelial cells, and hematopoietic cells isolated by fluorescence‐activated cell sorting (FACS) (Figure 3a). CD31 was used as a marker for vascular and lymphatic endothelial cells (Baluk & McDonald, 2008), and CD45 as a pan‐hematopoietic marker; no PDGFRα‐positive cells were detected within these cell groups (Figure 3a). Uniform Manifold Approximation and Projection (UMAP) analysis of PDGFRα‐positive cells revealed nine distinct clusters (Figure 3b, left). The expression patterns of marker genes associated with potential myofibroblast progenitor cells (Li et al., 2013; Li et al., 2017; Lua & Asahina, 2016; Tulasi et al., 2021; Yang et al., 2021) enabled us to attribute these clusters primarily to HSCs and PFs (Figure 3b, right). Specifically, the HSC marker gene *Lrat* was highly expressed in cluster 8, *Gucy1a1* in clusters 3, 4, 5, and 8, and *Des* in clusters 2, 3, 5, 6, and 8. PF marker genes were less selective than those for HSCs, but *Thy1* was highly expressed in clusters 0 and 6, *Slit2* in clusters 1 and 6, and *Eln* in cluster 1. In contrast, expression of mesothelial and cholangiocyte marker genes was nearly absent in PDGFRα‐positive cells (Figure 3b). These findings indicate that, despite challenges in classifying PFs using existing marker genes, PDGFRα‐positive cells in the adult mouse liver are primarily composed of HSCs and PFs. Fibroblasts in several organs can be broadly categorized into those that produce abundant extracellular matrix (ECM) and those that mediate interactions with immune cells, with *Dpp4* and *Pi16* as markers for the former and *Col15a1* and *Cxcl14* for the latter (Buechler et al., 2021; Oprescu et al., 2020; Patrick et al., 2024). Consistent with this, PFs and HSCs were divided into *Dpp4*‐positive and *Cxcl14*‐positive populations, respectively (Figure 3c). While no significant differences in *Pdgfra* expression levels were observed across clusters, *Col1a1* expression varied between clusters (Figure 3c). These results suggest that HSCs and PFs may be further subdivided into cell populations with distinct immune response characteristics.

### 2.4 Analysis of cluster‐specific interactions between PDGFRα‐positive and CD45‐positive cells

**FIGURE 4:**
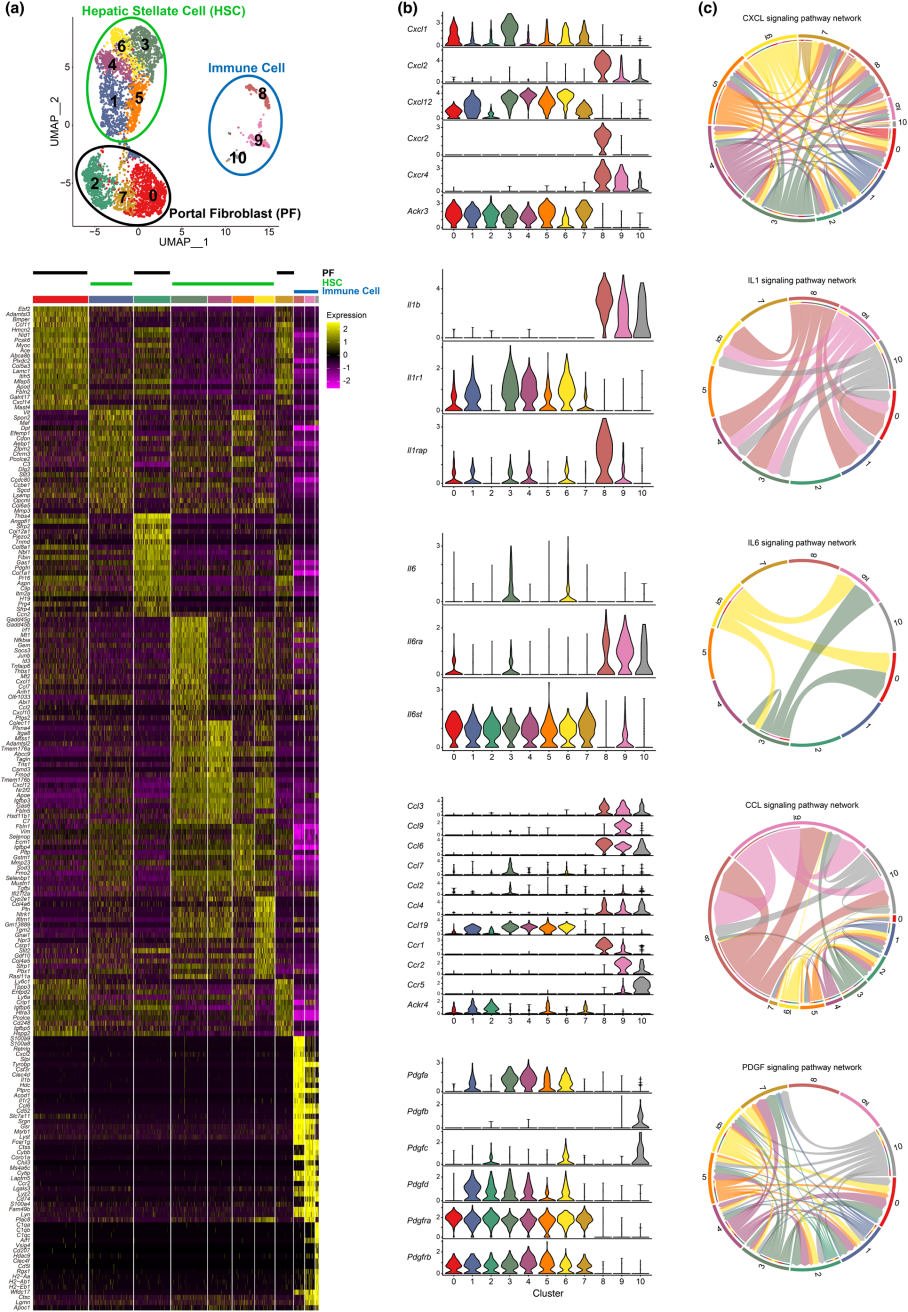
Single‐cell RNA sequencing reveals that a specific subtype of PDGFRα‐positive cells engages in ligand‐receptor interactions with CD45‐positive cells. (a) UMAP plot shows PDGFRα‐positive and CD45‐positive cells from mouse liver based on single‐cell RNA‐seq data (upper). PDGFRα‐positive cells are clustered in 0–7, and CD45‐positive cells are clustered in 8–10. Heatmap displays the top 20 genes highly expressed in each cluster (downer). (b, c) Results of inter‐cluster communication analysis using CellChat. Violin plots (b) represent the expression of gene sets associated with CXCL, IL1, IL6, CCL, and PDGF signaling pathway for each cluster. The chord diagrams (c) show the inter‐cluster interactions in each signaling pathway.

Based on the accumulation of PDGFRα‐positive and CD45‐positive cells in specific liver regions during fibrosis, as shown in Figures 1 and 2, and the scRNA‐seq results for PDGFRα‐positive cells in healthy livers, as shown in Figure 3, we hypothesized that subtypes of PDGFRα‐positive cells capable of selectively interacting with immune cells exist in the healthy liver. To explore potential cell–cell interactions, we performed CellChat analysis using the scRNA‐seq data from Figure 3, focusing on interactions between PDGFRα‐positive and CD45‐positive cells. PDGFRα‐positive cells were divided into eight clusters (clusters 0–7), and CD45‐positive cells into three clusters (clusters 8–10) (Figure 4a). Among CD45‐positive cells, cluster 8, expressing *Cd14*, likely represents monocytes, while clusters 9 and 10, expressing *Adgre1*, are primarily composed of macrophages or Kupffer cells (Figure S3). CellChat analysis of CXCL, IL1, IL6, and CCL signaling pathways revealed, as expected, that PDGFRα‐positive cell clusters exhibit varying capacities for interaction with immune cells (Figure 4b,c). Notably, cluster 3 within the PDGFRα‐positive cell population was identified as having the potential to interact with CD45‐positive cells through the highest number of signaling pathways. PDGF signaling is known to regulate cell proliferation, migration, and survival, contributing to fibrosis progression by promoting fibroblast resistance to apoptosis and cell division (Klinkhammer et al., 2018). Interestingly, the ligand *Pdgfa* was highly expressed exclusively in clusters defined as HSCs (Figure 4c, bottom). Consistent with previous studies indicating that PDGF‐B, which promotes HSC proliferation and significantly contributes to fibrosis, is selectively expressed in infiltrating macrophages (Borkham‐Kamphorst & Weiskirchen, 2016), *Pdgfb* expression was observed only in cluster 10 (Figure 4c, bottom). In line with the established use of PDGFRβ as a marker for liver fibroblasts, *Pdgfrb* expression was detected across all PDGFRα‐positive cell clusters (Figure 4c, bottom). These findings indicate that PDGFRα‐positive cells, comprising HSCs and portal fibroblasts (PFs), can be further subdivided based on their reactivity with immune cells. Specifically, we identified the potential existence of a PDGFRα‐positive cell subtype that selectively interacts with immune cells through multiple signaling pathways.

### 2.5 Localization of COX2‐positive PDGFRα‐positive cells around bile ducts

**FIGURE 5:**
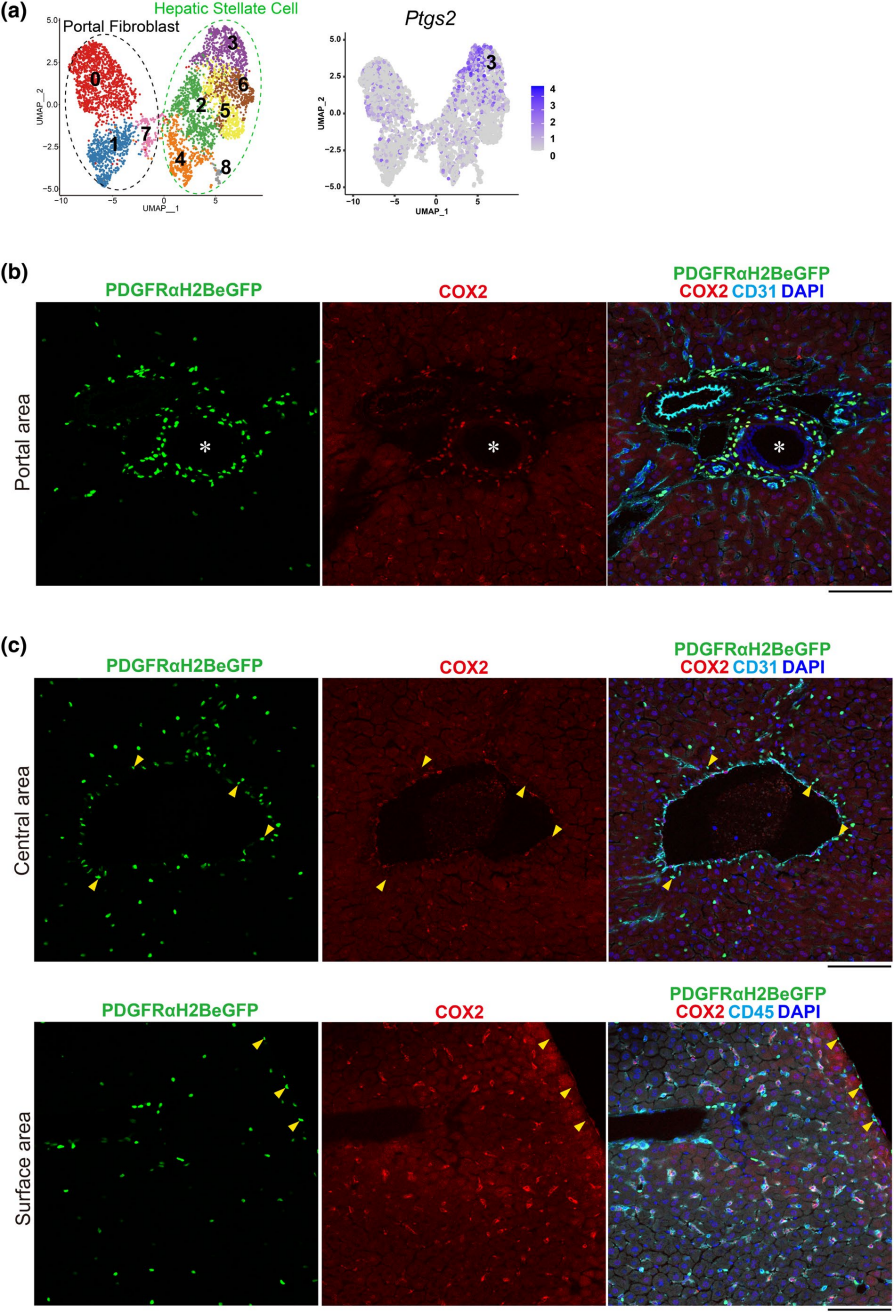
A COX2‐positive subtype of PDGFRα‐positive cells interacting with immune cells resides around the bile ducts. (a) The feature plot for *Ptgs2*. (b, c) Images of frozen sections from PDGFRα^EGFP^ mouse livers stained for COX2 (red), DAPI (blue), CD31 or CD45 (cyan), and PDGFRα^EGFP^ (green). Asterisk shows the bile duct of mouse liver. Scale bar = 100 μm.

To further characterize the PDGFRα‐positive cells in cluster 3, identified in Figure 4 as potentially interacting selectively with immune cells, we investigated the marker genes specific to this cluster. To identify genes specific to cluster 3, we compared gene expression levels between cluster 3 and other clusters, extracting the top 20 genes with the highest log2 fold change (log2FC) (Table S3). From these, we selected *Ptgs2* as a gene encoding a non‐secreted protein, with low expression (≤30%) in clusters other than cluster 3 (Figure 4a, Suppl. Table 4). *Ptgs2* is the gene encoding the COX2 protein. In lung fibrosis research, which has progressed rapidly alongside liver fibrosis studies, the presence of COX2‐positive PDGFRα‐positive cells that interact with immune cells has been reported (Gong et al., 2022). Feature plot confirmed that *Ptgs2* was enriched in cluster 3 PDGFRα‐positive cells (Figure 5a). Immunostaining revealed that COX2‐positive PDGFRα‐positive cells were localized around bile ducts (Figure 5b). In contrast, PDGFRα‐positive cells located around central veins were COX2‐negative (Figure 5c, yellow arrowheads). In addition, COX2‐positive cells located on the liver surface were CD45‐positive hematopoietic cells, while PDGFRα‐positive cells in this region were consistently COX2‐negative (Figure 5c, yellow arrowheads). The location of bile ducts was verified using cytokeratin 19 staining, a bile duct cell marker, and hematoxylin and eosin (H&E) staining on serial sections (Figure S4A,B). Furthermore, newly acquired four‐color confocal images (PDGFRα, COX2, CK19, and DAPI) from an independent sample confirmed the peri‐biliary localization of COX2‐positive PDGFRα‐positive fibroblasts (Figure S4C). Together, these findings demonstrate that COX2‐positive PDGFRα‐positive fibroblasts, which may selectively interact with immune cells, are specifically enriched in the peri‐biliary niche of the liver, whereas PDGFRα‐positive cells in pericentral regions or on the liver surface lack COX2 expression.

### 2.6 IL‐1β stimulation enhances COX2 expression in PDGFRα‐positive cells in vitro

**FIGURE 6:**
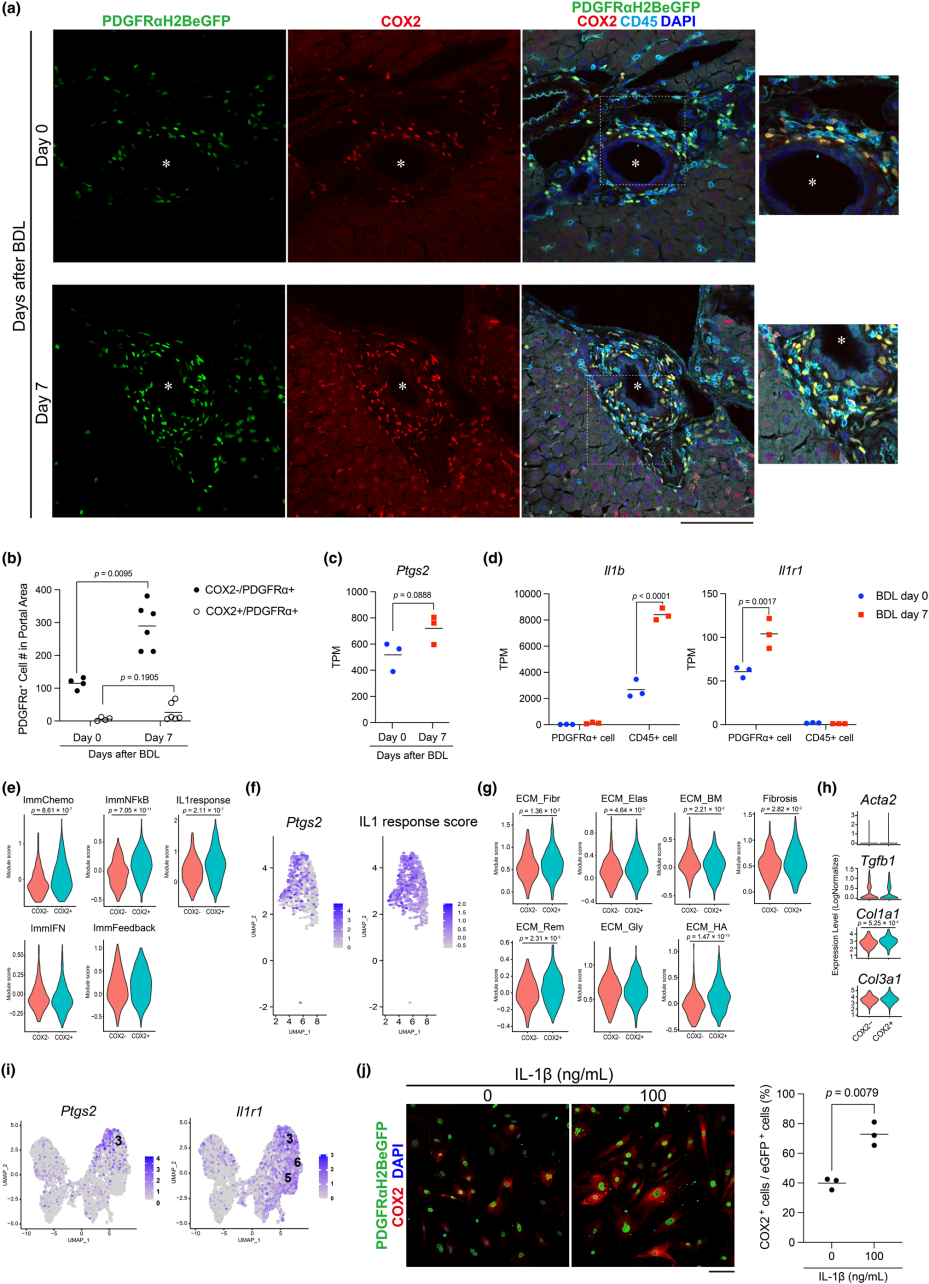
IL‐1β induces primary cultured PDGFRα‐positive cells to express COX2. (a) Images of frozen sections from PDGFRα^EGFP^ mouse livers stained for COX2 (red), DAPI (blue), and CD45 (cyan) for day 0 and day 7 after the BDL operation. The images on the right are enlarged views of the area enclosed by the white dashed line in the images on the left. Asterisk shows the bile duct of mouse liver. Scale bar = 100 μm. (b) Quantification of COX2‐positive cells among PDGFRα^EGFP^‐positive cells at Day 0 and 7 after BDL. Each dot represents one mouse (Day 0, *n* = 4; Day 7, *n* = 6). One image per mouse was analyzed. Exact *p*‐values are indicated in the graph; statistical analysis was performed using the Mann–Whitney *U* test. (c, d) TPM values of *Ptgs2* (C) and *Il1b*, *Il1r1*. Exact *p*‐values are indicated. (d) from the bulk RNA‐seq data shown in Figure 2c. (e) Violin plots showing module scores of immune response programs (ImmChemo, ImmNFkB, ImmIFN, ImmFeedback, and IL1 response) in COX2^+^ versus COX2^−^ cells within cluster 3 from the scRNA‐seq data shown in Figure 3. *p*‐values for the COX2 status term, adjusted for total RNA counts per cell (nCount_RNA) using linear regression models, are indicated in the graphs. (f) Feature plots of *Ptgs2* (left) and IL1 response module score (right) projected onto the UMAP embedding of cluster 3 cells from the scRNA‐seq data shown in Figure 3. (g) Violin plots showing module scores of extracellular matrix (ECM) categories (ECM_Fibr, ECM_Elas, ECM_BM, ECM_Rem, ECM_Gly, ECM_HA) and fibrosis/HSC activation (Fibrosis) in COX2^+^ versus COX2^−^ cells within cluster 3. *p*‐values for the COX2 status term, adjusted for total RNA counts per cell (nCount_RNA) using linear regression models, are indicated in the graphs. (h) Violin plots showing expression levels of key fibrosis‐related genes (*Acta2*, *Tgfb1*, *Col1a1*, *Col3a1*) in COX2^+^ versus COX2^−^ cells within cluster 3. *p*‐values for the COX2 status term, adjusted for total RNA counts per cell (nCount_RNA) using linear regression models, are indicated in the graphs. (i) Feature plots of *Ptgs2* and *Il1r1* from the scRNA‐seq data shown in Figure 3. (j) Images of PDGFRα‐positive cells isolated from the livers of two PDGFRαEGFP mice and cultured with or without IL‐1β for 12 h are shown on the left. Cells were stained for COX2 (red) and DAPI (blue). The graph on the right shows the percentage of COX2‐positive cells among PDGFRαEGFP‐positive cells. Each dot represents one well (*n* = 3 wells per group). For quantification, two images were acquired from each well and averaged to obtain a single value. The bars in the graphs represent the mean value of the samples (b–d and j). Scale bar = 100 μm (a and j).

We next examined COX2 expression in PDGFRα‐positive cells under fibrotic conditions. Immunostaining revealed a high abundance of COX2‐positive PDGFRα‐positive cells surrounding bile ducts at day 7 after bile duct ligation (BDL) (Figure 6a). Although the proportion of COX2‐positive cells among PDGFRα‐positive cells showed a trend toward increase after BDL, this did not reach statistical significance (*p* = 0.1905) (Figure 6b). Reanalysis of publicly available scRNA‐seq datasets (Yang et al., 2021) revealed a higher proportion of COX2‐positive PDGFRα‐positive cells in fibrotic livers from both BDL‐ and CCl4‐treated mice compared with healthy controls (control: 0.26%, BDL: 1.4%, CCl4: 2.2%; Figure S5A–D). Consistently, bulk RNA sequencing of FACS‐isolated PDGFRα‐positive cells showed a trend toward increased *Ptgs2* expression at day 7 post‐BDL compared with healthy controls (*p* = 0.0888) (Figure 6c). Immunostaining further demonstrated dense co‐localization of COX2‐positive PDGFRα‐positive cells with CD45‐positive immune cells around bile ducts during fibrosis (Figure 6a). Based on this spatial association, we hypothesized that cytokines secreted by CD45‐positive immune cells during fibrosis may promote COX2 expression in PDGFRα‐positive cells. To explore potential mediators of this crosstalk, we examined ligand–receptor pairs upregulated in CD45‐positive and PDGFRα‐positive cells at Day 7 post‐BDL. Among candidate cytokines previously shown to be upregulated in hematopoietic cells after BDL (*Il1b*, *Il6*, *Tgfb1*, *Pdgf*, and *Tnf*), the *Il1b*–*Il1r1* pair exhibited the most prominent expression changes (Figure 6d, Figure S6).

We therefore performed a detailed scRNA‐seq analysis focusing on cluster 3, the PDGFRα‐positive cell cluster exhibiting the highest *Ptgs2* expression, to characterize the transcriptional state of COX2‐positive cells. Within this cluster, COX2‐positive cells showed robust enrichment of inflammatory immune‐response programs, including chemokine signaling, NF‐κB–associated pathways, and an IL‐1–responsive transcriptional program (Figure 6e), whereas interferon‐related gene programs were not globally induced. UMAP feature plots further showed overlapping expression of *Ptgs2* and IL‐1 response scores within cluster 3 (Figure 6f). Analysis of extracellular matrix–associated gene programs revealed modest but significant differences between COX2‐positive and COX2‐negative cells; however, these differences were dominated by ECM remodeling and hyaluronan‐associated pathways, rather than classical fibrillar collagen or basement membrane programs (Figure 6g). Importantly, canonical markers of hepatic stellate cell activation and profibrotic signaling, including *Acta2* and *Tgfb1*, were not significantly upregulated, and expression of key fibrillar collagen genes (*Col1a1* and *Col3a1*) was only modestly altered (Figure 6h). Consistent with previous reports showing that the inflammatory cytokine IL‐1β induces COX2 expression in lung fibroblasts (Gong et al., 2022), single‐cell RNA sequencing revealed high expression of Il1r1 in clusters 3, which include COX2‐positive PDGFRα‐positive cells (Figure 6i).

Finally, to determine whether this IL‐1–responsive COX2^+^ state could be recapitulated in vitro, PDGFRα‐positive cells isolated from healthy mouse livers were treated with IL‐1β for 12 h. IL‐1β stimulation significantly increased the proportion of COX2‐expressing cells (Figure 6j). Notably, IL‐1β treatment did not significantly alter the αSMA‐positive cell area in cultured PDGFRα‐positive cells (Figure S7). Together, these findings indicate that PDGFRα^+^ COX2^+^ cells primarily adopt an inflammation‐associated fibroblast‐like state downstream of IL‐1 signaling, rather than a classical HSC activation or overt fibrogenic phenotype.

## 3 DISCUSSION

This study, using a bile duct ligation (BDL) mouse model, revealed that in cholestatic liver fibrosis, the number of PDGFRα‐positive fibroblasts selectively increases around bile ducts from the early stages of the disease, accompanied by an accumulation of immune cells in the same region. scRNA‐seq analysis demonstrated that the PDGFRα‐positive fibroblast population primarily consists of hepatic stellate cells and portal fibroblasts. Furthermore, we identified COX2‐positive PDGFRα‐positive fibroblasts, which likely engage in selective crosstalk with immune cells, specifically localized around bile ducts.

Causes of human liver fibrosis include viral infections, excessive intake of chemicals such as alcohol, cholangitis, and metabolic disorders (Kisseleva & Brenner, 2021). To model these conditions, animal models of liver disease are generated using methods such as carbon tetrachloride (CCl_4_) administration, bile duct ligation (BDL), or high‐fat diet feeding (Wu et al., 2023). The choice of model influences the progression of fibrosis and the response to therapeutic interventions. In the BDL mouse model used in this study, PDGFRα‐positive fibroblasts accumulated around bile ducts following surgery (Figure 1a). In contrast, prior studies have reported that in the CCl4 administration model, PDGFRα‐positive fibroblasts predominantly accumulate near central veins (Hayes et al., 2014), consistent with the distinct sites of injury in these models. One factor contributing to the variability in experimental outcomes across liver fibrosis models may be the diversity of PDGFRα‐positive fibroblast populations identified in this study. Single‐cell analyses from prior studies have shown that the composition of fibroblast populations varies across different liver fibrosis models (Yang et al., 2021), suggesting that the PDGFRα‐positive cell populations responding to liver injury may differ depending on the disease model. Previous research using IL‐1Ra knockout mice, which lack the IL‐1β antagonist, demonstrated that BDL in these mice resulted in increased fibrosis compared to wild‐type controls, whereas CCl4 administration in IL‐1Ra knockout mice led to reduced fibrosis (Meier et al., 2019). These observations suggest that model‐dependent differences in fibrotic responses may arise from the selective activation of distinct IL‐1β‐responsive fibroblast populations, including subsets of PDGFRα‐positive cells, rather than differences in IL‐1β signaling intensity alone.

PDGFRα^EGFP^ mice exhibit reduced PDGFRα expression, and previous studies have demonstrated that these mice develop attenuated liver fibrosis compared with wild‐type mice (Hayes et al., 2014). In addition, it has been reported that HSC‐specific *Pdgfra* knockout mice show fibrosis comparable to that in wild‐type mice in the bile duct ligation (BDL)‐induced liver fibrosis model, whereas fibrosis progression is attenuated specifically during the early to middle stages in the CCl₄‐induced model (Kikuchi et al., 2020). In the present study, we observed distinct regional differences in the proliferation of PDGFRα‐positive cells between wild‐type and PDGFRα^EGFP^ mice following BDL. In wild‐type mice, PDGFRα‐positive cells increased significantly even in non–peri‐biliary regions by day 7 post‐BDL, whereas in PDGFRαEGFP mice the increase remained largely confined to peri‐biliary regions. This regional difference could reflect differences in PDGFRα signaling thresholds between peri‐biliary and non–peri‐biliary regions, potentially due to variations in baseline or injury‐induced PDGFRα expression levels and/or local PDGF ligand availability. Thus, peri‐biliary PDGFRα‐positive cells may retain sufficient signaling capacity to drive proliferation even under reduced PDGFRα expression, whereas PDGFRα‐positive cells in non–peri‐biliary regions (including hepatic stellate cells) may fall below the threshold required for comparable responses. Such zone‐dependent heterogeneity in PDGF responsiveness could help explain the model‐specific effects of PDGFRα attenuation reported across studies and warrants further investigation using zone‐specific transcriptomic approaches or PDGF ligand localization analyses.

In this study, we identified COX2 (*Ptgs2*) as a marker for a subtype of PDGFRα‐positive fibroblasts that likely interacts selectively with immune cells (Figure 5). We also demonstrated that these cells may increase around bile ducts during liver fibrosis, with hematopoietic cell‐derived IL‐1β potentially contributing to their proliferation (Figure 6). COX2 is an enzyme that catalyzes the synthesis of prostaglandins, inflammatory mediators, from arachidonic acid and is implicated in both inflammation and cell proliferation (Sobolewski et al., 2010). COX2‐positive PDGFRα‐positive fibroblast subtypes have been reported in various organs. In the colon, COX2‐positive PDGFRα‐positive cells are localized at the apex of intestinal crypts (Muhl et al., 2020), and in a dextran sulfate sodium‐induced colitis mouse model, *Ptgs2* knockout mice exhibited impaired epithelial proliferation and tissue regeneration in crypts compared to wild‐type mice (Brown et al., 2007). In the lung, COX2‐positive PDGFRα‐positive cells in the adventitial space have been reported to regulate immune responses (Gong et al., 2022). In the liver, COX2 expression is known to increase in various cell types during inflammation or cancer, and its expression has been observed in hepatic stellate cells of cirrhosis patients (Hu et al., 2020; Mohammed et al., 2004). Regarding the relationship between IL‐1β and COX2 expression in fibroblasts, co‐culture of lung fibroblasts with immune cells increased *Ptgs2* expression, which was suppressed by IL‐1β inhibitors (Gong et al., 2022). In the heart, fibroblasts increase in response to immune cell‐derived IL‐1β, potentially regulating the progression of heart disease (Amrute et al., 2024). These findings suggest that COX2‐positive PDGFRα‐positive fibroblasts, located in specific tissue niches across various organs, may play a critical role in regulating organ fibrosis progression through interactions with immune cells.

## 4 LIMITATION

This study has several limitations that should be acknowledged. First, single‐cell RNA sequencing was performed only using cells isolated from healthy livers. Direct analysis of PDGFRα‐positive fibroblasts from fibrotic livers was technically challenging because PDGFRα expression was reduced after fibrosis induction, and enzymatic dissociation of fibrotic tissue caused substantial cellular damage due to dense extracellular matrix deposition. Consequently, transcriptional dynamics during fibrosis progression could not be directly evaluated. Second, although IL‐1β‐induced COX2 upregulation was demonstrated in vitro, in vivo functional perturbation experiments were not performed, and causal relationships during fibrosis progression therefore remain to be established. Third, this study exclusively employed the bile duct ligation model, which represents cholestatic liver injury. Since fibroblast activation states and spatial distributions differ across fibrosis models, the generalizability of the identified COX2‐positive, PDGFRα‐positive fibroblast subset to other forms of liver fibrosis remains unclear. Future studies using single‐nucleus RNA sequencing approaches for fibrotic tissues, combined with genetic labeling and FACS‐based enrichment strategies for rare fibroblast populations, will be important to further validate and extend these findings.

## 5 METHODS

### 5.1 Mice

C57BL/6J male and female mice were purchased from Shiraishi Animals Co., Ltd. (Saitama, Japan). PDGFRα^EGFP^ mice (stock# 007669), which are maintained on a C57BL/6J genetic background, were purchased from The Jackson Laboratory (Bar Harbor, ME, USA). The mice were fed a standard pellet diet (MF, Oriental Yeast Co., Ltd., Tokyo, Japan). They were supplied with food and water ad libitum and kept at a constant temperature and humidity under a 12‐h dark/light cycle. Male and female mice were used and distributed equally among experimental groups. All animal experiments were approved by the Experimental Animal Care and Use Committee of the University of Tokyo.

### 5.2 BDL model

Mice were randomly divided into the control (Day 0) group and the BDL group. Mice in the BDL group were anesthetized with isoflurane, and their abdomens were surgically opened. The common bile duct was then ligated at two points using thread, followed by closure. On Days 0, 4, or 7 post‐surgery, mice were deeply anesthetized with isoflurane and euthanized by cervical dislocation, after which the livers were harvested for experiments.

### 5.3 Histology

Livers for histological analysis were snap‐frozen in isopentane chilled with liquid nitrogen and sectioned into 10‐μm‐thick sections using a cryostat (# CM1860, Leica Biosystems, Heidelberger, Germany). The sections were fixed with 4% paraformaldehyde for 5 min, washed three times with phosphate‐buffered saline (PBS), and blocked with Blocking One (# 03953‐66, Nacalai Tesque, Kyoto, Japan) for 10 min. The samples were incubated with primary antibodies overnight at 4°C. The following day, after washing three times with PBS, the sections were incubated with secondary antibodies at room temperature for 1 h, loaded with DAPI for 5 min. After three additional PBS washes, they were mounted using SlowFade™ Diamond Antifade Mountant (# S36972, Thermo Fisher Scientific, Waltham, MA, USA). For whole‐mount staining, a small piece of liver was fixed in 4% paraformaldehyde (PFA) for 30 min and incubated with first antibody and second antibody for 24 h each. Confocal fluorescence images were acquired using an A1Rsi high‐speed laser scanning confocal microscope (Nikon Instruments Inc.), which was mounted on a motorized inverted fluorescence microscope (Ti‐E) equipped with a Perfect Focus System (PFS). Images were obtained using a 10x or 20x objective lens. Image acquisition and processing were performed using NIS‐Elements software (NIS‐C ER; Nikon Instruments Inc.). Quantification of Collagen1 or CD45‐positive area and counting of eGFP‐positive cells were performed using WinROOF2021 (Version 5.3.0, Mitani Corporation, Fukui, Japan). The primary and secondary antibodies used for staining are listed in Suppl. Table 5. Some of the slides were subjected to cover glass removal after observation, followed by hematoxylin and eosin staining.

### 5.4 Cell isolation

Mice were anesthetized and their chests were opened. A 23G needle was inserted into the heart, and the liver was perfused with 20 mL of pre‐warmed (37°C) 0.05% collagenase type 2 (# LS004176, Worthington Biochemical Corporation, Lakewood, NJ, USA) in DMEM at a controlled flow rate not exceeding 2 mL/min to preserve cell viability. The liver was harvested and chopped into small pieces, followed by digestion with 10 mL of 0.1% collagenase type 2 and 2.4 U/mL Dispase II (# 17105‐041, Thermo Fisher Scientific) in DMEM at 37°C for 1 h. The liver tissue was passed through 18G and 21G needles five times each, diluted up to 100 mL with PBS, and filtered through 100 μm and 40 μm cell strainers. The suspension was centrifuged at 1000×*g* for 5 min, the supernatant was discarded, and the cell pellet was resuspended with 600 μL of washing buffer (2.5% FBS in PBS). After incubating with antibodies at 4°C for 30 min, the suspension was diluted to 1 mL with washing buffer and centrifuged. The antibodies used for staining are listed in Suppl. Table 5. The supernatant was discarded again, and the cell pellet was resuspended in washing buffer containing SYTOX® Red dead cell stain (1:1000, REF: S34859, Life Technologies, Carlsbad, CA, USA). The gating strategy was as follows: first, FSC‐A versus SSC‐A was used to exclude debris. Next, FSC‐A versus FSC‐H was used to select single cells. From the singlet population, SYTOX‐negative cells were gated as living cells. Subsequent gating steps used to isolate specific cell populations are shown in Figure 1d, while the complete gating workflow is provided in Figure S8. For sample collection for scRNA‐seq, Actinomycin D (Cat# A1410‐5MG, Sigma‐Aldrich, St. Louis, MO, USA) was added at a concentration of 20 μM to the digestion solution and 2 μM to the subsequent suspension.

### 5.5 RNA extraction and real‐time PCR analysis

Total RNA was extracted from sorted cells using RNeasy Micro Kit (# 74004, QIAGEN, Venlo, Nederland). The extracted total RNA was reverse‐transcribed into cDNA using QuantiTect Reverse Transcription Kit (# 205313, QIAGEN). Real‐time PCR was conducted with THUNDERBIRD Next SYBR qPCR Mix (# QPX‐201, TOYOBO, Osaka, Japan). The PCR conditions were as follows: 95°C for 30 s, followed by 40 cycles of 95°C for 5 s and 60°C for 30 s. The relative expression was quantified by ΔΔ CT method. *Cma*s was used as a reference gene. For real‐time PCR, the following primers were used: *Cmas* Forward: 5′‐GACTCTGTCTTCTCCGTTGTGAG‐3′ *Cmas* Reverse: 5′‐ACTCTCCATCCCAGTCTTGTCG‐3′ *Col1a1* Forward: 5′‐CCGAACCCCAAGGAAAAGAA‐3′ *Col1a1* Reverse: 5′‐GTGGACATTAGGCGCAGGAA‐3′.

### 5.6 Bulk RNA sequence

Total RNA was extracted from sorted cells using the RNeasy Micro Kit (QIAGEN). Library construction was performed according to the provided instructions using the Smart‐seq v4 Ultra Low Input RNA Kit for Sequencing (# 634888, Takara Bio, Shiga, Japan) and Nextera TX DNA Library Prep Kit for Illumina (# FC‐131‐1024, Illumina, San Diego, CA, USA). Libraries were sequenced using Illumina NovaSeq 6000 (Illumina), and paired‐end sequencing reads were aligned to the mouse reference genome (GPCm38) using DRAGEN Bio‐IT Platform (version 3.6.3, Illumina). The subsequent data analysis was performed using iDEP (version 2.01) (Ge et al., 2018). DEGs were identified using DESeq2 with an FDR cutoff of 0.1 and a minimum fold‐change of 2, followed by the generation of volcano plots and GO analysis.

### 5.7 Single‐cell RNA sequence

Using flow cytometry, PDGFRα‐positive cells, CD31‐positive cells, and CD45‐positive cells were isolated and mixed in a ratio of 3:1:1. The total number of cells was adjusted to 10,000, and this mixture was used to construct a scRNA‐seq library. The following reagents were used according to the instructions for library preparation: Dual Index Kit NN Set A (# 1000243, 10× Genomics, Pleasanton, CA, USA), Chromium Next GEM Single Cell 3’ Kit v3.1 (# 1000268, 10× Genomics), Chromium Next GEM Chip G Single Cell Kit (# 1000127, 10× Genomics). Libraries were sequenced using Illumina NovaSeq X Plus (Illumina). The following scRNA‐seq analysis was performed using SCALA (Tzaferis et al., 2022), a graphical user interface–based analysis platform. Within SCALA, data preprocessing, normalization, dimensionality reduction, clustering, and differential gene expression analyses were conducted using Seurat (v4) with default parameters unless otherwise specified. Cell–cell communication analysis, including the generation of chord diagrams, was performed using CellChat (v1) with default settings.

Publicly available scRNA‐seq datasets from BDL‐ and CCl4‐induced mouse liver fibrosis models reported by Yang et al. (Yang et al., 2021) were obtained from the Gene Expression Omnibus (GEO) under accession number GSE171904 and reanalyzed using the same SCALA/Seurat‐based workflow described above. For reanalysis of publicly available datasets, cells expressing *Pdgfra* >1.0 and *Ptgs2* >1.0 were defined as *Pdgfra*/*Ptgs2* double‐positive cells. The proportion of *Ptgs2*‐positive cells among *Pdgfra*‐positive cells was calculated for each dataset.

### 5.8 Differential analysis of module scores and gene expression in COX2 ^+^ vs. COX2 ^−^ cells within cluster 3

Within cluster 3, cells were classified as COX2‐positive (COX2^+^) or COX2‐negative (COX2^−^) based on detectable expression of *Ptgs2* (raw counts >0). This binary classification was used consistently for all comparative analyses. To characterize transcriptional programs associated with inflammation, extracellular matrix (ECM) organization, and fibrogenesis, predefined gene sets were assembled based on established literature and functional annotations. Complete gene lists for all modules are provided in Table S6. Differences in module scores and gene expression levels between COX2^+^ and COX2^−^ cells were assessed using linear regression models, with COX2 status included as the main explanatory variable and total RNA counts per cell (nCount_RNA) included as a covariate to account for potential confounding effects of sequencing depth. *p*‐values for the COX2 status term were extracted from each model and are reported in the Figure. Statistical significance was defined as *p* < 0.05.

### 5.9 Cell culture and IL‐1β treatment

Sorted cells were seeded onto a Matrigel‐coated (Matrigel® Growth Factor Reduced (GFR) Basement Membrane Matrix, LDEV‐free, REF:354230, Corning, NY, USA) 96 well plate (# 6055300, PerkinElmer, Waltham, MA, USA). The cells were cultured in growth medium (DMEM supplemented with 20% FBS, 1% penicillin–streptomycin, and 2.5 ng/mL basic fibroblast growth factor) (REF:16005293, Hygieia Bioscience, Osaka, Japan) at 37°C in 5% CO2. After reaching 80% confluency, the cells were incubated with 100 ng/mL recombinant mouse IL‐1β (# 401‐ML/CF, R&D Systems, Minneapolis, MN, USA) for 12 h. Cells were fixed in 4% PFA for 5 min and permeabilized with 0.1% triton X‐100. Subsequently, cells were blocked and incubated with the first antibody at 4°C overnight, followed by incubation with the second antibody for 1 h at room temperature. Images were captured using the Nikon A1RSi and COX2‐positive areas were quantified using WinROOF2021.

### 5.10 Statistical analyses

All statistical analyses were performed using Prism (Version 10.3.0, GraphPad Software, San Diego, CA, USA). The difference in mean values between two groups was assessed using an unpaired *t*‐test with Welch's correction. The homogeneity of variance among three groups was assessed using the Brown‐Forsythe test, and differences in mean values among the three groups were analyzed using a one‐way analysis of variance. Differences were considered statistically significant when the *p* value was < 0.05. The effect size for each study was estimated based on our previous study (Kurosawa et al., 2021) and preliminary experimental data. The sample sizes were determined using Sample Size Calculations from the IACUC at Boston university for the type I error rate = 0.05 and power = 0.8. No data show significant deviation from normal distribution and no data were excluded in this study.

## AUTHOR CONTRIBUTIONS

**Momo Goto:** Conceptualization; data curation; formal analysis. **Tamaki Kurosawa:** Conceptualization; funding acquisition; project administration; resources; supervision. **Hiroyuki Koike:** Data curation; methodology. **Noriyuki Kaji:** Data curation; methodology. **Ichiro Manabe:** Methodology; software. **Taiki Mihara:** Funding acquisition; methodology. **Takashi Chaen:** Data curation; methodology. **Yumiko Oishi:** Funding acquisition; methodology; resources. **Masatoshi Hori:** Funding acquisition; methodology; resources.

## FUNDING INFORMATION

This work was supported by Grants‐in‐Aid for Scientific Research (KAKENHI) (23KJ0510 to M.G.; 22KJ2353 to T.K.; 22K15008 and 24K18016 to T.M.; 23H02912 to Y.O.; 19H03125 and 23H02379 to M.H.), the grant of The University of Tokyo R4 Excellent Young Researcher to T.K., the grant of Japan Science and Technology Agency (JST, grant number JPMJFR2351 to Y.O.), and the grant of Takeda Science Foundation to Y.O.

## CONFLICT OF INTEREST STATEMENT

The authors declare no conflicts of interest.

## ETHICS STATEMENT

All animal experiments were approved by the Experimental Animal Care and Use Committee of the University of Tokyo.

## Supporting information


**Table S1.** DESeq2 differential expression analysis of PDGFRα^+^ cells between BDL Day 0 and 7 as shown in Figure 2c.

**Table S2.** DESeq2 differential expression analysis of CD45^+^ cells between BDL Day 0 and 7 as shown in Figure 2c.

**Table S3.** Differentially expressed genes in cluster 3 from scRNA‐seq analysis.

**Figure S1.** BDL surgery in wild‐type mice increases the PDGFRα‐positive area in the liver.
**Figure S2.** Expression of Cxcl1 and Cxcr2 in PDGFRα‐positive cells and CD45‐positive cells.
**Figure S3.** Feature plots of immune cell marker genes.
**Figure S4.** Visualization of Bile ducts in stained mouse liver serial sections.
**Figure S5.** Reanalysis of publicly available scRNA‐seq datasets from mouse liver fibrosis models.
**Figure S6.** TPM values of ligand and receptor genes associated with inflammation and fibrosis in PDGFRα‐positive and CD45‐positive cells.
**Figure S7.** IL‐1β does not alter αSMA‐positive cell area in PDGFRα‐positive cells.
**Figure S8.** Full flow cytometric gating strategy for isolation of liver cell populations.
**Table S4.** Top 20 differentially expressed genes in cluster 3.
**Table S5.** List of antibodies used in this study.
**Table S6.** Gene sets and references used for module score analysis.

## Data Availability

The scRNA‐seq data of this study are openly available in the database DDBJ at (https://www.ddbj.nig.ac.jp), reference number DRR728309‐DRR728312. The bulk RNA‐seq data are also available in the database DDBJ, reference number DRR727849‐DRR727860. All the data presented here are available from the corresponding authors upon reasonable request.
