# Supplementary material for: COX2 +/PDGFRα + fibroblasts selectively localize near bile ducts and interact with immune cells in early liver fibrosis in mice
Source: Physiol Rep. 2026 May 24;14(10):e70938. doi: 10.14814/phy2.70938 (PMC13240249; doi:10.14814/phy2.70938)
Supplement: Supplementary file 4 — Figure S1. BDL surgery in wild‐type mice increases the PDGFRα‐positive area in the liver. Figure S2. Expression of Cxcl1 and Cxcr2 in PDGFRα‐positive cells and CD45‐positive cells. Figure S3. Feature plots of immune cell marker genes. Figure S4. Visualization of Bile ducts in stained mouse liver serial sections. Figure S5. Reanalysis of publicly available scRNA‐seq datasets from mouse liver fibrosis models. Figure S6. TPM values of ligand and receptor genes associated with inflammation and fibrosis in PDGFRα‐positive and CD45‐positive cells. Figure S7. IL‐1β does not alter αSMA‐positive cell area in PDGFRα‐positive cells. Figure S8. Full flow cytometric gating strategy for isolation of liver cell populations. Table S4. Top 20 differentially expressed genes in cluster 3. Table S5. List of antibodies used in this study. Table S6. Gene sets and references used for module score analysis. [file PHY2-14-e70938-s004.pdf]

Supplemental Information

Momo Goto et al.

COX2^{+}/PDGFRα^{+} fibroblasts selectively localize near bile ducts and interact with immune cells in early liver fibrosis in mice

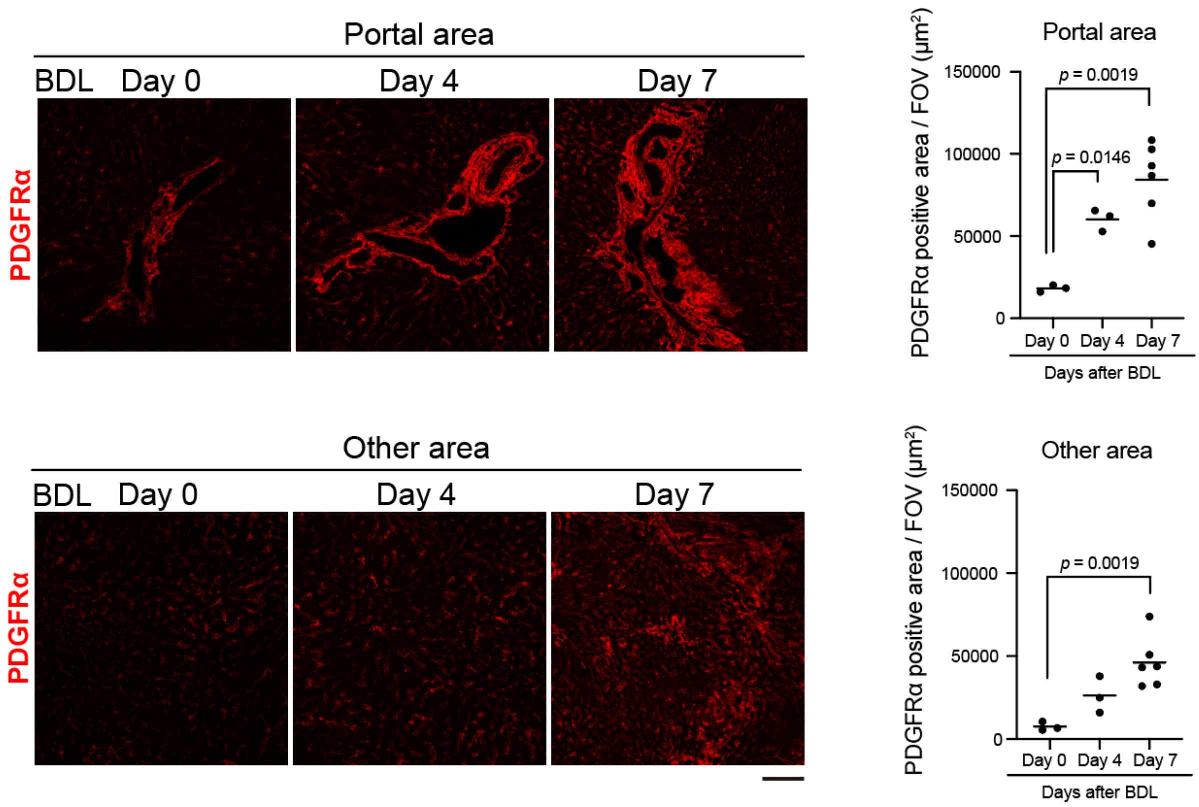

Supplemental Figure 1: BDL surgery in wild-type mice increases the PDGFRα-positive area in the liver.

Images of frozen sections from WT mouse livers stained for PDGFRα (red). The images are identical to those shown in Fig. 1B. Quantification of the PDGFRα-positive area was performed using the same images. Each dot represents one mouse (day 0 and day 4, $n = 3$; day 7, $n = 6$). For each mouse, two different fields from tissue sections were imaged and averaged to obtain a single value, which is shown as one dot. Bars indicate the mean value for each group. Scale bar = 100 μm.

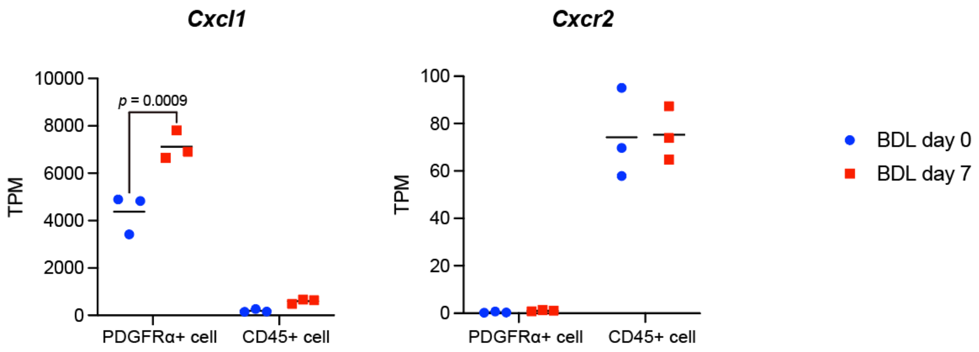

Supplemental Figure 2: Expression of *Cxcl1* and *Cxcr2* in PDGFRα-positive cells and CD45-positive cells.

Graphs show TPM values for *Cxcl1* and *Cxcr2* obtained from bulk RNA-seq of PDGFRα-positive cells and CD45-positive cells isolated from mouse livers before and after BDL. Each dot represents one mouse ($n = 3$). The bars represent the mean expression levels across samples.

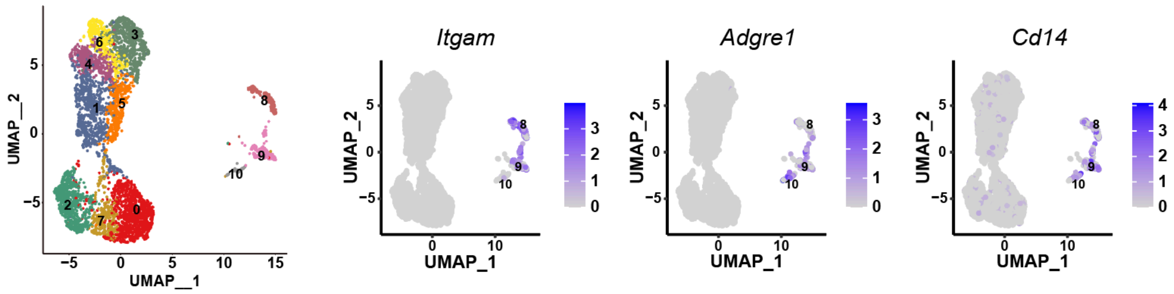

Supplemental Figure 3: Feature plots of immune cell marker genes.

UMAP plot displays PDGFRα-positive cells and CD45-positive cells from mouse liver based on single-cell RNA-seq data (left). Feature plots display the expression of *Itgam*, *Adgre1*, and *Cd14* in PDGFRα-positive cells and CD45-positive cells (right).

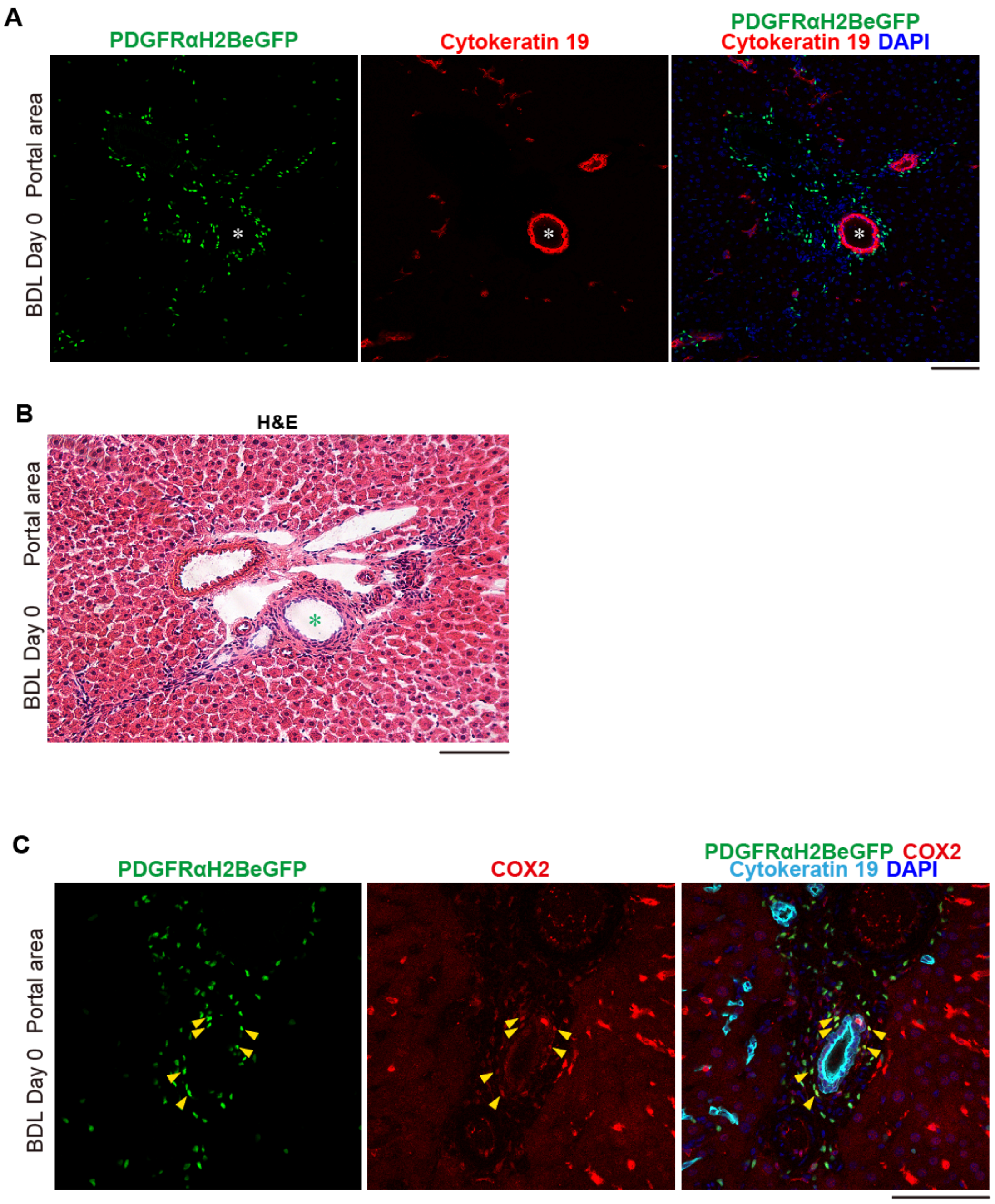

Supplemental Figure 4: Visualization of Bile ducts in stained mouse liver serial sections.

Images in Fig. 5B and Supplementary Fig. 3A–B represent serial sections, with asterisks indicating the same bile duct across sections. (A) Frozen liver sections from PDGFRα^{EGFP} mice stained for cytokeratin 19 (CK19, red) and DAPI (blue) before BDL surgery, showing bile duct localization corresponding to Fig. 5B. (B) Hematoxylin and eosin (H&E) staining of a serial section adjacent to panel A, confirming tissue architecture. (C) Representative four-color confocal image from an independent PDGFRα^{EGFP} mouse liver sample stained for PDGFRα (green), COX2 (red), CK19 (light blue), and DAPI (blue), demonstrating the peri-biliary localization of COX2-positive PDGFRα-positive fibroblasts using CK19 as a bile duct marker. Yellow arrowheads indicate PDGFRα/COX2 double-positive cells. Scale bar = 100 μm.

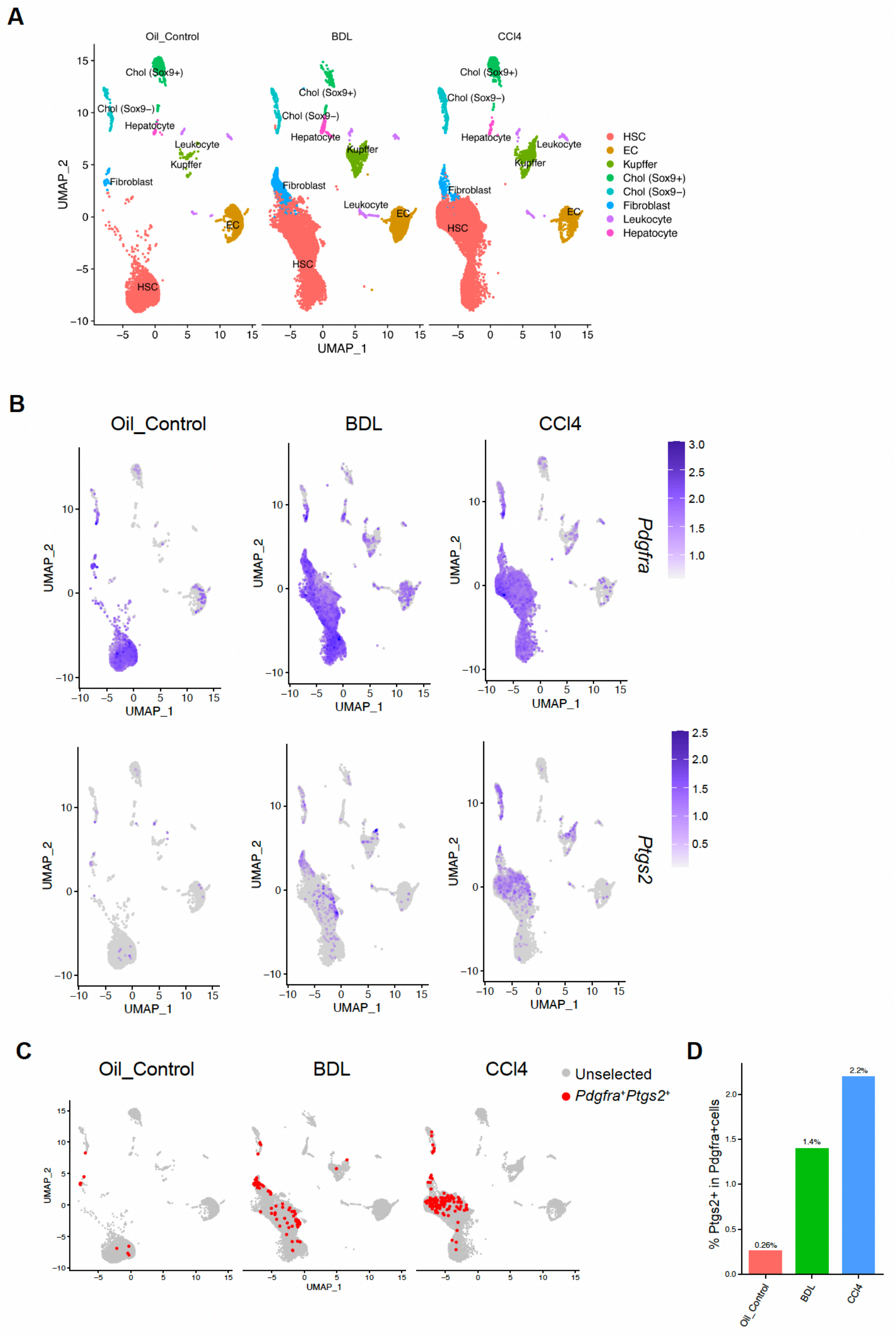

Supplemental Figure 5: Reanalysis of publicly available scRNA-seq datasets from mouse liver fibrosis models.

(A) UMAP visualization showing annotated cell populations from Oil_Control, bile duct ligation (BDL), and CCl4-treated mouse livers. (B) Feature plots showing expression of Pdgfra and Ptgs2 in each dataset. (C) Cells co-expressing *Pdgfra* and *Ptgs2* were defined using expression thresholds (Pdgfra > 1.0 and Ptgs2 > 1.0) and are highlighted in red on the UMAP plots. Gray dots indicate unselected cells. (D) Quantification of the proportion of *Ptgs2*-positive cells among *Pdgfra*-positive cells in each condition.

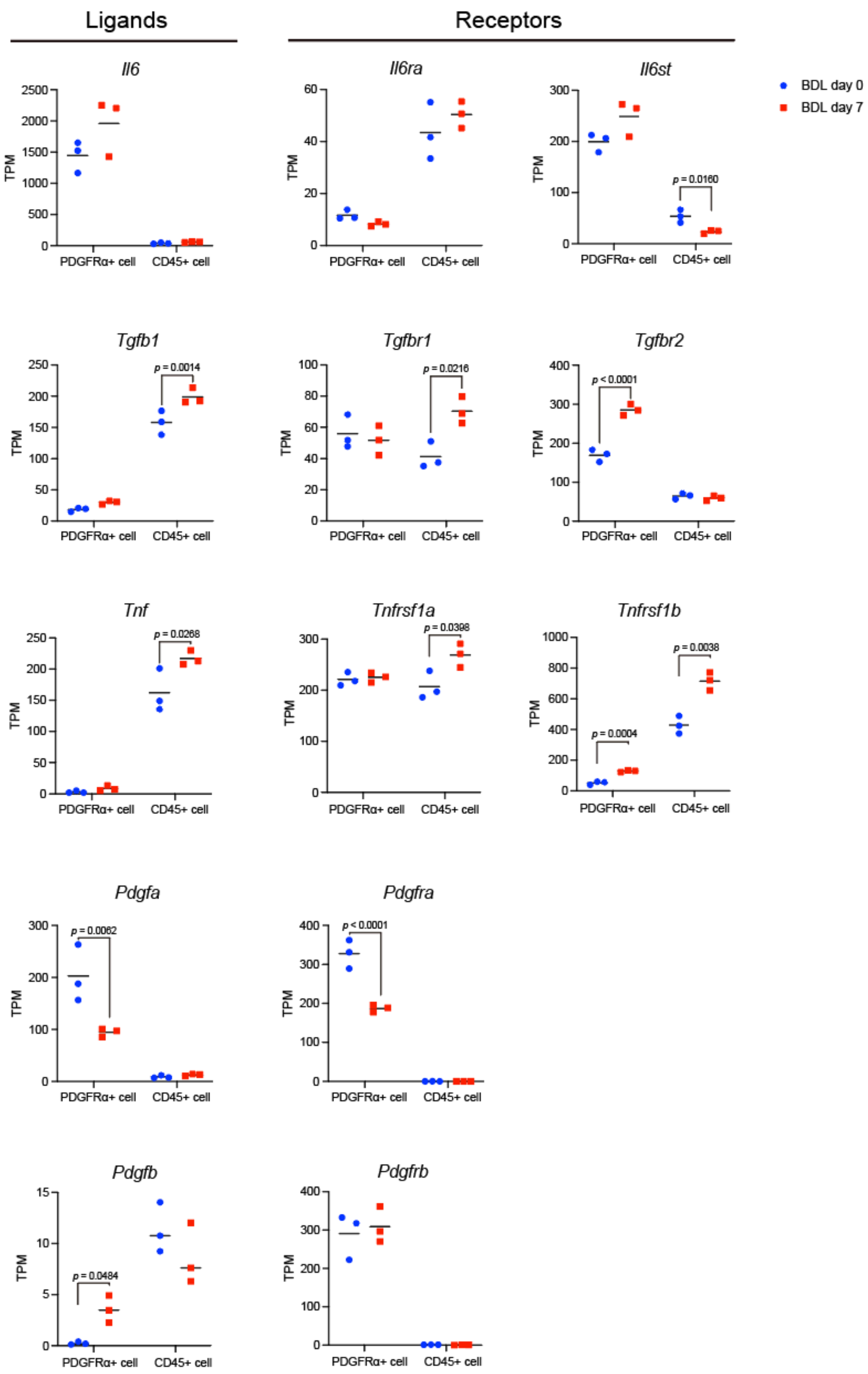

Supplemental Figure 6: TPM values of ligand and receptor genes associated with inflammation and fibrosis in PDGFRα-positive and CD45-positive cells.

Graphs show TPM values for 13 ligand and receptor genes associated with inflammation and fibrosis, obtained from bulk RNA-seq of PDGFRα-positive cells and CD45-positive cells isolated from mouse livers before and after BDL operation. Each dop represents one mouse ($n = 3$). The bars represent mean expression levels across samples.

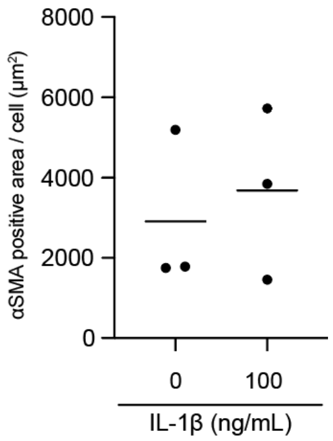

Supplemental Figure 7: IL-1β does not alter αSMA-positive cell area in PDGFRα-positive cells

PDGFRα-positive cells FACS-sorted from the livers of two PDGFRα^{EGFP} mice were treated with or without IL-1β for 12 hours. Cells were stained for αSMA antibody, and the αSMA-positive area was quantified. Each dot represents one field. The bars represent the mean of three fields.

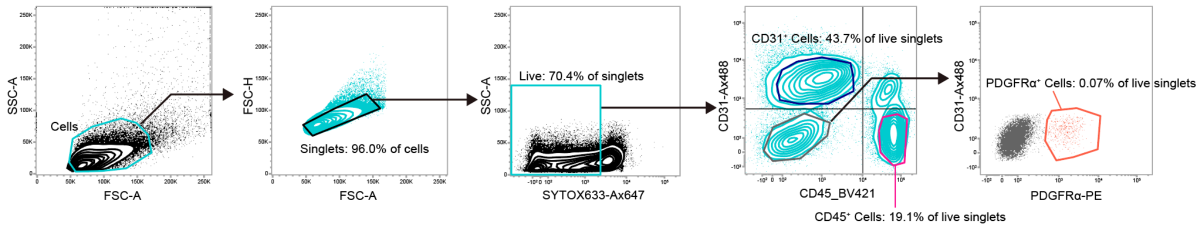

Supplemental Figure 8: Full flow cytometric gating strategy for isolation of liver cell populations.

Representative flow cytometric gating strategy used for the isolation of liver cell populations. Cells were first gated based on forward scatter (FSC-A) versus side scatter (SSC-A) to exclude debris. Single cells were then selected using FSC-A versus FSC-H to remove doublets. From the singlet population, live cells were identified as SYTOX-negative. Subsequently, live singlets were gated to identify the indicated cell populations, including PDGFRα-positive, CD31-positive, and CD45-positive cells, which were used for downstream analyses. Percentages shown in each plot indicate the frequency of cells relative to the parent gate.

Supplemental Table 1: DESeq2 differential expression analysis of PDGFRα^{+} cells between BDL day 0 and day 7 as shown in Figure 2C.

Differential gene expression was assessed by DESeq2 using bulk RNA-seq data. For each gene included in the analysis, log2 fold change and adjusted p-value (padj) are reported. This data is provided separately as a Excel file.

Supplemental Table 2: DESeq2 differential expression analysis of CD45^{+} cells between BDL day 0 and day 7 as shown in Figure 2C.

Differential gene expression was assessed by DESeq2 using bulk RNA-seq data. For each gene included in the analysis, log2 fold change and adjusted p-value (padj) are reported. This data is provided separately as a Excel file.

Supplemental Table 3: Differentially expressed genes in cluster 3 from scRNA-seq analysis

This is a list of genes selectively expressed in cluster 3 of the scRNA-seq data shown in Fig. 5A. Genes are ranked in ascending order of adjusted p-values (p_val_adj). This data is provided separately as a Excel file.

p_val	avg_log2FC	pct.1	pct.2	p_val_adj	Cluster	Gene	Location (Uniprot Annotation)
4.58E-88	1.672432626	0.37	0.097	9.72E-84	3	*Cxcl10*	secreted
1.27E-89	2.042614906	0.378	0.099	2.70E-85	3	*Ccl2*	secreted
8.66E-138	2.511342119	0.541	0.151	1.84E-133	3	*Ccl7*	secreted
1.18E-252	2.462687062	0.821	0.244	2.50E-248	3	*Gadd45g*	No UniProt annotations available.
1.16E-63	1.757483315	0.555	0.276	2.45E-59	3	***Ptgs2***	microsome membrane, endoplasmic reticulum membrane, nucleus inner membrane, nucleus outer membrane
2.44E-154	1.984753802	0.793	0.337	5.17E-150	3	*Thbs1*	secreted, cell surface, endoplasmic reticulum
4.97E-230	1.633506232	0.888	0.365	1.05E-225	3	*Irf1*	nucleus, cytoplasm
1.53E-143	2.184288895	0.832	0.423	3.25E-139	3	*Cxcl1*	secreted
1.00E-202	1.727027102	0.938	0.462	2.13E-198	3	*Socs3*	No UniProt annotations available.
2.68E-151	1.758767688	0.899	0.516	5.68E-147	3	*Mt2*	No UniProt annotations available.
3.82E-248	2.270849708	0.97	0.518	8.10E-244	3	*Gadd45b*	No UniProt annotations available.
1.94E-122	1.697123769	0.913	0.551	4.11E-118	3	*Olfr1033*	cell membrane
4.72E-208	1.643101565	0.961	0.554	1.00E-203	3	*Nfkbia*	cytoplasm, nucleus
8.08E-159	2.048691691	0.916	0.607	1.71E-154	3	*Tnfaip6*	secreted
2.07E-133	1.717072365	0.914	0.634	4.38E-129	3	*Arih1*	cytoplasm, nucleus, nucleus (Cajal body)
1.21E-205	1.690485364	0.991	0.711	2.57E-201	3	*Gem*	cell membrane
6.96E-101	1.808169902	0.928	0.742	1.48E-96	3	*Abi1*	cytoplasm, nucleus, cell projection, postsynaptic density
5.71E-194	1.955756967	0.997	0.802	1.21E-189	3	*Id3*	nucleus, cytoplasm
3.58E-209	2.44806674	0.988	0.81	7.60E-205	3	*Mt1*	No UniProt annotations available.
6.36E-196	1.979419267	0.997	0.837	1.35E-191	3	*Junb*	nucleus

Supplemental Table 4: Top 20 differentially expressed genes in cluster 3

The table lists the top 20 differentially expressed genes in cluster 3 from scRNA-seq analysis, ranked in descending order by log_2 fold change. Subcellular location data retrieved from the UniProt database are included.

Supplemental Table 5: List of antibodies used in this study

Application	Product Name	Manufacturer	Cat. Number	Host	Dilution
Immunostaining	Anti-Cytokeratin 19 Antibody, clone TROMA-3	Sigma-Aldrich	MABT913	Rat	1 : 500
	Alexa Fluor® 488 anti-mouse CD45 Antibody	Biolegend	103122	Rat	1 : 300
	CD45 Monoclonal Antibody (30-F11), APC-eFluor™ 780, eBioscience™	Invitrogen	47-0451-82	Rat	1 : 300
	Mouse PDGF R alpha Antibody	R&D Systems	AF1062	Goat	1 : 400
	Anti-Collagen I antibody	Abcam	ab21286	Rabbit	1 : 200
	BD Pharmingen™ Purified Rat Anti-Mouse CD31	BD Pharmingen	557355	Rat	1 : 200
	Cox2 (D5H5) XP® Rabbit mAb	Cell Signaling	12282	Rabbit	1 : 400
	Anti-GFP antibody	Abcam	ab13970	Chicken	1 : 1000
	Donkey anti-Rabbit IgG (H+L) Highly Cross-Adsorbed Secondary Antibody, Alexa Fluor™ 488	Invitrogen	A21206	Donkey	1 : 1000
	Donkey anti-Rabbit IgG (H+L) Highly Cross-Adsorbed Secondary Antibody, Alexa Fluor™ 594	Invitrogen	A21207	Donkey	1 : 1000
	Donkey anti-Rabbit IgG (H+L) Highly Cross-Adsorbed Secondary Antibody, Alexa Fluor™ 647	Invitrogen	A31537	Donkey	1 : 1000
	Donkey anti-Goat IgG (H+L) Cross-Adsorbed Secondary Antibody, Alexa Fluor™ 594	Invitrogen	A11058	Donkey	1 : 1000
	Alexa Fluor® 647 AffiniPure™ Donkey Anti-Goat IgG (H+L)	Jackson Immunoresearch	705-605-147	Donkey	1 : 1000
	Goat anti-Chicken IgY (H+L) Secondary Antibody, Alexa Fluor™ 488	Invitrogen	A11039	Goat	1 : 1000
	Donkey anti-Rat IgG (H+L) Highly Cross-Adsorbed Secondary Antibody, Alexa Fluor™ 488	Invitrogen	A21208	Donkey	1 : 1000
	Donkey anti-Rat IgG (H+L) Highly Cross-Adsorbed Secondary Antibody, Alexa Fluor™ 594	Invitrogen	A21209	Donkey	1 : 1000
	Alexa Fluor® 647 AffiniPure™ Donkey Anti-Rat IgG (H+L)	Jackson Immunoresearch	712-605-153	Donkey	1 : 1000
Cell sorting	Mouse PDGF R alpha PE-conjugated Antibody	R&D Systems	FAB1062P	Goat	1 : 60
	Alexa Fluor® 488 anti-mouse CD31 Antibody	Biolegend	102414	Rat	1 : 300
	Brilliant Violet 421™ anti-mouse CD45 Antibody	Biolegend	103134	Rat	1 : 300
	Brilliant Violet 421™ anti-mouse CD31 Antibody	Biolegend	102424	Rat	1 : 300
	CD45 Monoclonal Antibody (30-F11), APC-eFluor™ 780, eBioscience™	Invitrogen	47-0451-82	Rat	1 : 300

Supplemental Table 6: Gene sets and references used for module score analysis

A. List of genes comprising the module scores used for differential analysis of module scores and gene expression between COX2^{+} and COX2^{-} cells within cluster 3.

Category	Module	Pathway_annotation	Key_references	Genes
ECM	ECM_BM	Basement membrane components	Theocharis et al., 2016; Frantz et al., 2010	Col4a1, Col4a2, Col4a3, Col4a4, Col6a1, Col6a2, Col6a3, Hspg2, Lama1, Lama2, Lamb1, Lamb2, Lamc1, Nid1, Nid2
ECM	ECM_Elas	Elastic fiber / microfibril-associated genes	Bonnans et al., 2014	Efemp1, Eln, Fbln1, Fbln2, Fbln5, Fbn1, Fbn2, Mfap2, Mfap4, Mfap5
ECM	ECM_Fibr	Fibrillar collagens / interstitial ECM	Karsdal et al., 2017; Theocharis et al., 2016	Col11a1, Col12a1, Col14a1, Col15a1, Col1a1, Col1a2, Col3a1, Col5a1, Col5a2
ECM	ECM_Gly	Proteoglycans / glycoproteins / matricellular	Theocharis et al., 2016; Frantz et al., 2010	Bgn, Dcn, Fn1, Lum, Postn, Sdc1, Sdc2, Sdc4, Sparc, Thbs1, Thbs2, Tnc, Vcan
ECM	ECM_HA	Hyaluronan metabolism	Jiang et al., 2011	Has1, Has2, Has3, Hyal1, Hyal2, Tnfaip6, Ugdh
ECM	ECM_Rem	ECM remodeling (MMP/TIMP/LOX/serpins)	Bonnans et al., 2014; Rockey et al., 2015	Adamts2, Adamts4, Adamts5, Lox, Loxl1, Loxl2, Mmp14, Mmp2, Mmp9, Plau, Plaur, Serpine1, Timp1, Timp2
Fibrosis	Fib	HSC activation / profibrotic signaling & ECM	Tsuchida & Friedman, 2017; Kisseleva & Brenner, 2021; Friedman, 2008	Acta2, Cnn1, Col1a1, Col1a2, Col3a1, Ctgf, Lox, Serpine1, Tagln, Tgfb1, Timp1
Immune	IL1response	IL-1 receptor signaling / NFkB and MAPK-AP-1 immediate early response	Dinarello, 2009; Weber et al., 2010; Mantovani et al., 2019	Ccl2, Cxcl1, Cxcl2, Fos, Il6, Junb, Nfkbia, Tnfaip3
Immune	ImmChemo	Chemokine signaling / leukocyte recruitment	Zlotnik & Yoshie, 2000; Zlotnik & Yoshie, 2012	Ccl2, Ccl3, Ccl4, Ccl5, Ccl7, Ccl8, Cxcl1, Cxcl10, Cxcl11, Cxcl2, Cxcl3, Cxcl9
Immune	ImmFeedback	Cytokine/MAPK feedback regulators	Kubo et al., 2003; Lang et al., 2019; Jadhav et al., 2017; Kovarik et al., 2021	Atf3, Cish, Dusp1, Dusp5, Socs1, Socs3, Zfp36
Immune	ImmIFN	Type I/II interferon signaling / ISG response	Ivashkiv & Donlin, 2014; Schneider et al., 2014	Bst2, Ifit1, Ifit2, Ifit3, Irf1, Irf7, Isg15, Mx1, Rsad2, Stat1, Stat2, Usp18
Immune	ImmNFkB	TNFalpha, NFkB / inflammatory signaling	Luedde & Schwabe, 2011; Sunami et al., 2012	Il6, Nfkb1, Nfkb2, Nfkbia, Nfkbiz, Nos2, Ptges, Rel, Rela, Tnf, Tnfaip2, Tnfaip3

B. References supporting the selection of gene sets used for module score calculation.

Module	Pathway_annotation	Authors	Title	Year	URL
ECM_BM, ECM_Gly, ECM_Fibr	basement membrane components/proteoglycans / glycoproteins / matricellular/fibrillar collagens / interstitial ECM	Theocharis AD, et al	Extracellular matrix structure	2016	https://pubmed.ncbi.nlm.nih.gov/26562801/
ECM_BM, ECM_Gly	basement membrane components/proteoglycans / glycoproteins / matricellular	Frantz C, et al	The extracellular matrix at a glance	2010	https://pubmed.ncbi.nlm.nih.gov/21123617/
ECM_Elas, ECM_Rem	elastic fiber / microfibril-associated genes	Bonnans C, et al	Remodelling the extracellular matrix in development and disease	2014	https://pubmed.ncbi.nlm.nih.gov/25415508/
ECM_Fibr	fibrillar collagens / interstitial ECM	Karsdal MA, et al	The good and the bad collagens of fibrosis – Their role in signaling and organ function	2017	https://pubmed.ncbi.nlm.nih.gov/28736303/
ECM_HA	hyaluronan metabolism	Jiang D, et al	Hyaluronan as an immune regulator in human diseases	2011	https://pubmed.ncbi.nlm.nih.gov/21248167/
ECM_Rem	ECM remodeling: MMP/TIMP/LOX/serpins	Rockey DC, et al	Fibrosis—a common pathway to organ injury and failure	2015	https://pubmed.ncbi.nlm.nih.gov/25785971/
Fib	HSC activation / profibrotic signaling & ECM	Tsuchida T, Friedman SL	Mechanisms of hepatic stellate cell activation	2017	https://pubmed.ncbi.nlm.nih.gov/28487545/
Fib	HSC activation / profibrotic signaling & ECM	Kisseleva T, Brenner D	Molecular and cellular mechanisms of liver fibrosis and its regression	2021	https://pubmed.ncbi.nlm.nih.gov/33128017/
Fib	HSC activation / profibrotic signaling & ECM	Friedman SL	Mechanisms of hepatic fibrogenesis	2008	https://pubmed.ncbi.nlm.nih.gov/18471545/
IL1 response	IL-1 receptor signaling / NFkB and MAPK-AP-1 immediate early response	Weber A, Wasiliew P, Kracht M	Interleukin-1 (IL-1) pathway	2010	https://pubmed.ncbi.nlm.nih.gov/20086235/
IL1 response	IL-1 receptor signaling / NFkB and MAPK-AP-1 immediate early response	Dinarello CA	Immunological and inflammatory functions of the interleukin-1 family	2009	https://pubmed.ncbi.nlm.nih.gov/19302047/
IL1 response	IL-1 receptor signaling / NFkB and MAPK-AP-1 immediate early response	Mantovani A, et al	Interleukin-1 and Related Cytokines in the Regulation of Inflammation and Immunity 2019	2019	https://pubmed.ncbi.nlm.nih.gov/30995499/
ImmChemo	Chemokine signaling / leukocyte recruitment	Zlotnik A, Yoshie O	Chemokines: a new classification system and their role in immunity	2000	https://pubmed.ncbi.nlm.nih.gov/10714678/
ImmChemo	Chemokine signaling / leukocyte recruitment	Zlotnik A, Yoshie O	The Chemokine Superfamily Revisited	2012	https://pubmed.ncbi.nlm.nih.gov/22633458/
ImmFeedback	Cytokine/MAPK feedback regulators	Kubo M, et al	Suppressors of cytokine signaling and immunity	2003	https://pubmed.ncbi.nlm.nih.gov/14639467/
ImmFeedback	Cytokine/MAPK feedback regulators	Lang R, et al	Dual-Specificity Phosphatases in Immunity and Infection	2019	https://pubmed.ncbi.nlm.nih.gov/31159473/
ImmFeedback	Cytokine/MAPK feedback regulators	Jadhav K, et al	Activating transcription factor 3 in immune response and metabolic regulation	2017	https://pubmed.ncbi.nlm.nih.gov/29242753/
ImmFeedback	Cytokine/MAPK feedback regulators	Kovarik P, et al	Conceptual Advances in Control of Inflammation by the RNA-Binding Protein Tristetraprolin	2021	https://pubmed.ncbi.nlm.nih.gov/34603339/
ImmIFN	Type I/II IFN–STAT / ISG response	Ivashkiv LB, Donlin LT	Regulation of type I interferon responses	2014	https://pubmed.ncbi.nlm.nih.gov/24362405/
ImmIFN	Type I/II IFN–STAT / ISG response	Schneider WM, Chevillotte MD, Rice CM	Interferon-stimulated genes: a complex web of host defenses	2014	https://pubmed.ncbi.nlm.nih.gov/24555472/
ImmNFkB	TNFalpha, NFkB / inflammatory signaling	Luedde T, Schwabe RF	NF-κB in the liver—linking injury, fibrosis and hepatocellular carcinoma	2011	https://pubmed.ncbi.nlm.nih.gov/21293511/
ImmNFkB	TNFalpha, NFkB / inflammatory signaling	Sunami Y, et al	Hepatic activation of IKK/NF-κB signaling induces liver fibrosis via macrophage-mediated chronic inflammation	2012	https://pubmed.ncbi.nlm.nih.gov/22407857/
